# The Roles of Long-Term Hyperhomocysteinemia and Micronutrient Supplementation in the *App^NL–G–F^* Model of Alzheimer’s Disease

**DOI:** 10.3389/fnagi.2022.876826

**Published:** 2022-04-26

**Authors:** Hendrik Nieraad, Natasja de Bruin, Olga Arne, Martine C. J. Hofmann, Nina Pannwitz, Eduard Resch, Sonja Luckhardt, Ann-Kathrin Schneider, Sandra Trautmann, Yannick Schreiber, Robert Gurke, Michael J. Parnham, Uwe Till, Gerd Geisslinger

**Affiliations:** ^1^Fraunhofer Institute for Translational Medicine and Pharmacology, Frankfurt am Main, Germany; ^2^Pharmazentrum Frankfurt/ZAFES, Institute of Clinical Pharmacology, Goethe University, Frankfurt am Main, Germany; ^3^EpiEndo Pharmaceuticals, Reykjavík, Iceland; ^4^Former Institute of Pathobiochemistry, Friedrich-Schiller-Universität Jena, Jena, Germany

**Keywords:** hyperhomocysteinemia, vitamin B deficiency, Alzheimer’s disease, amyloid betapeptides, memory and learning tests, proteomics

## Abstract

A causal contribution of hyperhomocysteinemia to cognitive decline and Alzheimer’s disease (AD), as well as potential prevention or mitigation of the pathology by dietary intervention, have frequently been subjects of controversy. In the present *in vivo* study, we attempted to further elucidate the impact of elevated homocysteine (HCys) and homocysteic acid (HCA) levels, induced by dietary B-vitamin deficiency, and micronutrient supplementation on AD-like pathology, which was simulated using the amyloid-based *App^NL–G–F^* knock-in mouse model. For this purpose, cognitive assessment was complemented by analyses of *ex vivo* parameters in whole blood, serum, CSF, and brain tissues from the mice. Furthermore, neurotoxicity of HCys and HCA was assessed in a separate *in vitro* assay. In confirmation of our previous study, older *App^NL–G–F^* mice also exhibited subtle phenotypic impairment and extensive cerebral amyloidosis, whereas dietary manipulations did not result in significant effects. As revealed by proximity extension assay-based proteome analysis, the *App^NL–G–F^* genotype led to an upregulation of AD-characteristic neuronal markers. Hyperhomocysteinemia, in contrast, indicated mainly vascular effects. Overall, since there was an absence of a distinct phenotype despite both a significant amyloid-β burden and serum HCys elevation, the results in this study did not corroborate the pathological role of amyloid-β according to the “amyloid hypothesis,” nor of hyperhomocysteinemia on cognitive performance. Nevertheless, this study aided in further characterizing the *App^NL–G–F^* model and in elucidating the role of HCys in diverse biological processes. The idea of AD prevention with the investigated micronutrients, however, was not supported, at least in this mouse model of the disease.

## Introduction

A causal contribution of the endogenous amino acid homocysteine (HCys) and its metabolites to neurodegenerative diseases has been discussed for years. Alzheimer’s disease (AD), especially sporadic AD which is the highly prevalent and dominant late-onset form (Mullane and Williams, 2019), has previously been shown to be associated with increased HCys levels (Lai et al., 2017). One hallmark of sporadic AD is a deficiency in vitamin B12 (Regland and McCaddon, 2019). As pointed out in previous reviews, B-vitamin deficiency, especially lack of vitamin B6, B12, and folate, is one factor amongst others that results in increased HCys levels, called hyperhomocysteinemia (HHCys) (Kim et al., 2018; Price et al., 2018). Consequently, the reduction of HCys levels using B-vitamin intervention offers an interesting option and has been discussed frequently with respect to potential mitigation of AD-related pathology. In order to test HCys-lowering as a possibly valuable preventative approach, it is crucial to examine the role of B-vitamin intervention at a very early stage of the disease. The initial phase of AD pathology is typically characterized by disrupted amyloid metabolism, a central hallmark of this neurodegenerative disease (Parvathy et al., 2001; Sasaguri et al., 2017). Amyloid-β (Aβ) peptides aggregate and build up characteristic extracellular plaques, which can be detected many years before the onset of the first cognitive symptoms (Bateman et al., 2012). Soluble Aβ oligomers have been suggested to be the major detrimental Aβ species, particularly at an early stage of the disorder, also entitled preclinical AD (Müller-Schiffmann et al., 2016; Ye et al., 2017).

In terms of animal research, the long asymptomatic phase of AD can be simulated by animal models based on modifications of the amyloid-β precursor protein (AβPP) (Zahs and Ashe, 2010). Many AβPP-based mouse models are available, however, transgenic models often pose the risk of artificial phenotypes due to an overexpression of AβPP. This pitfall has been overcome by the introduction of knock-in mouse models such as *App^NL–G–F^* (Saito et al., 2014). Humanization of the AβPP sequence and implementation of three targeted mutations (NL-Swedish, G-Arctic, and F-Beyreuther/Iberian) generates distinct amyloidosis that resembles human AD and simulates aspects of neuroinflammation (Saito and Saido, 2018). The *App^NL–G–F^* KI mice were suggested to exhibit 3-fold faster and greater AD pathology and cognitive abnormalities compared with *App^NL–F^* mice (Sasaguri et al., 2017). The *App^NL–G–F^* KI mice were suggested to show extensive pathology as early as 6 months (Mehla et al., 2019; Sato et al., 2021).

Additional HHCys can be induced in experimental animals, e.g., by feeding special diets, which are deficient in specific micronutrients that are essential in the context of the remethylation or transsulfuration of HCys (Nieraad et al., 2021b). Besides supplementation with B-vitamins, HCys remethylation may also proceed independently of vitamins with betaine in some tissues (Nieraad et al., 2021b). Dietary betaine supplementation with the goal of mitigation of HHCys, has also been the subject of former animal studies (Chai et al., 2013; Gupta et al., 2016). According to an international consensus statement, also other micronutrients, such as polyunsaturated fatty acids (PUFA), should be taken into account in terms of controlling plasma HCys as a modifiable risk factor for dementia in the elderly (Smith et al., 2018). PUFA might play a role in slowing cognitive decline, however, evidence is not consistent on this topic (Beydoun et al., 2014). It has been shown that PUFA, particularly eicosapentaenoic acid (EPA) and docosahexaenoic acid (DHA), are required for an adequate effect of B-vitamin treatment in cognitive decline and for preventing HCys-related deterioration of cortical Aβ load (Oulhaj et al., 2016; Hooper et al., 2017). Equally, B-vitamin status is thought to be important for PUFA-associated effects on cognition and therefore, future studies on the link between these micronutrients and cognitive outcomes were imperative (Jernerén et al., 2019).

The research questions raised in the present *in vivo* study were mostly oriented toward our previous work on this topic (Nieraad et al., 2020) and a potential contribution of experimentally induced HHCys to the AD-like pathology in the *App^NL–G–F^* model was the focus of the study. Additionally to HCys, we also considered its oxidative metabolite homocysteic acid (HCA), which has been suggested be the actual neurotoxic culprit, as summarized recently (Nieraad et al., 2021b). This hypothesis was tested and is reported in the *in vitro* part of the present article. Aside from potentially detrimental effects of HHCys, we were interested in effects by the *App^NL–G–F^* model itself and investigated dietary effects of different micronutrients on various readouts, comprising behavioral cognitive outcomes, as well as *ex vivo* parameters in whole blood, serum, CSF, and brain tissues from the mice. In our previous study, we detected almost no aggravation of AD-like pathology by HHCys in *App^NL–G–F^* KI mice, which proved to be a very subtle model for the disease. Consequently, for the present long-term animal study, several experimental details have been adapted, including the diets to some extent, procedures, and outcomes. Most of all, the duration of the study was nearly doubled, enabling the examination of WT and KI mice at a higher age of approximately 70 weeks, with the aim to examine the experimental variables on a more distinct AD-like pathology. Overall, the current exploratory study can be seen as a continuation and extension of our previous study on this topic (Nieraad et al., 2020).

In a recently published review paper (Nieraad et al., 2021b), we reviewed literature on HHCys and cognitive performance. The majority of the reviewed animal studies indeed revealed an impact of HHCys, meaning that, in these studies, at least one of the conducted behavioral tests showed significant effects following an elevation of HCys levels. Some examples of such studies showing impairments on cognition due to HHCys: a methyl-donor-free diet supplemented with homocysteine induced in aged APP-overexpressing mice (Bernardo et al., 2007), vitamin-B dietary deficiency in TgCRND8 mice (Fuso et al., 2008, 2012), in C57BL6/J mice (Troen et al., 2008), in Apolipoprotein-E (ApoE)-deficient mice (Troen et al., 2006), and HCys injections in Sprague-Dawley rats (Mahaman et al., 2018). It should, however, also be considered that negative results are often not published, although equally important as positive results. The publication bias, meaning the reduced publishing of negative or null results, is not restricted to the field of AD research, but is rather a general problem (Bespalov et al., 2019).

In addition, it is important to emphasize that a potential factor contributing to the failure to identify effective treatments for AD in preclinical studies, is the lack of consideration of other pathological mechanisms besides the Aβ pathology, as discussed in a comprehensive review (Banik et al., 2015). The authors propose that a more balanced approach should be used, since AD is a multifactorial disease. As shown in our previous study, despite prominent Aβ plaque deposition, the *App^NL–G–F^* mice merely displayed a very mild AD-like phenotype at the investigated age (Nieraad et al., 2020), which was the reason why we extended the duration of our present study. The same holds true for our investigation of the potential risk factor: despite the high levels of homocysteine in the blood due to the vitamin-B deficient diet, this did not result in significant effects on learning and memory performance in our previous study (Nieraad et al., 2020). Therefore, here we investigated the combined effects of amyloidosis, increased aging and long-term HHCys induced by a vitamin-B deficient diet.

## Materials and Methods

### Cell Viability and *in vitro* Assay

Examination of cell viability after compound treatment was performed using the luciferase-based CellTiter-Glo assay (Promega, Walldorf, Germany). Prior to the assay, primary cortical neurons (rat brain, R-CX-500, Lonza, Basel, Switzerland) were plated into a poly-D-lysine coated opaque 96-well plate (Corning, Wiesbaden, Germany). Therefore, counting of viable cells in the cell suspension was done with the help of trypan blue staining (Sigma-Aldrich). Approximately 10,000 cells were plated per well. Cultivation of the cells on the plate proceeded in Neurobasal medium, supplemented with B27 (minus antioxidants), L-glutamine, and penicillin-streptomycin (ThermoFisher, Frankfurt am Main, Germany) at 37°C, 5% CO2. To increase cell survival and reduce debris, the first 4 h of cell culture was done in a medium containing 5% fetal calf serum (ThermoFisher), which was subsequently replaced by serum-free medium. First neurite networks became visible by day 3. At day 4, 50% of the medium was changed. Finally, after 5 days of cell culture, the actual assay was performed on 2 consecutive days. Either the test compounds L-homocysteine or L-homocysteic acid (Sigma-Aldrich, Taufkirchen, Germany), celecoxib as a frequently used positive control for this purpose (Biomol, Hamburg, Deutschland) or vehicle were pipetted into the plate. Compound treatment wells (cells + test compound in different concentrations), positive control (cells + 10 mM celecoxib), negative control (cells + vehicle), and blank wells (medium without cells + vehicle) were fully randomized over the 96-well plate. After an incubation period of 24 h, an equal amount of CellTiter-Glo reagent was added to each well, shaken, and read out after 10 min at a luminometer (Perkin Elmer, Hamburg, Germany). The luminescence signal (relative light units) of blank wells was subtracted from all treatment and control wells. The signal of the negative control was defined as 100% cell viability. A luciferase-based assay was chosen, since other common testing systems based on water soluble tetrazolium or comparable substances were not applicable due to the redox properties of HCys, which would lead to an interaction with the assay reagent. We performed the present *in vitro* assay in *n* = 3 and furthermore used triplicates on each plate.

### Animals, Experimental Groups, and Diets

All animal experiments were carried out in accordance with the “3R,” the DIRECTIVE 2010/63/EU and the regulations of GV-SOLAS. Experimental procedures were approved by the local Ethics Committee for Animal Research in Darmstadt, Germany (approval number: F152/1011; approval date: 31.07.2017) and based on European Quality In Preclinical Data (EQIPD) and the ARRIVE-Guidelines (Bespalov et al., 2021).

In total, 84 mice were examined in the present study, equally consisting of males and females. Twelve age-matched C57BL/6J wild type (WT) animals were obtained from Charles River Wiga GmbH (Sulzfeld, Germany) and 72 homozygous *App^NL–G–F^* knock-in (KI) animals were bred at mfd Diagnostics GmbH (Wendelsheim, Germany). The *App^NL–G–F^* knock-in model was kindly provided by Dr. Saido and colleagues from RIKEN Center for Brain Science (Saitama, Japan). All animals were chipped with subcutaneous transponders in order to minimize the risk of erroneous animal allocation. Genotyping at our lab, using polymerase-chain-reaction analysis, confirmed the adequate genetic background of each animal. The mice were randomly assigned to their home cages (Green Line, Tecniplast, Hohenpeissenberg, Germany). All randomization steps in the current study were based on randomization lists.^1^ Animals were housed pairwise at constant temperature (mean: 22.7°C) and humidity (mean: 53.6%) conditions under a 12/12 h dark/light cycle (lights on at 7:00 a.m. with twilight phases starting at 6:30 a.m. and 18:30 p.m.). The use of sentinel mice enabled monitoring of the hygiene status of the colony room.

One week after arrival, the mice were allocated to seven experimental groups (Table 1). The seven groups were defined by the animals’ genotype and the different experimental diets that the KI animals received. Compositions of the diets were based on the AIN93M chow and modified as shown in detail in Supplementary Table 1. In comparison to our previous study (Nieraad et al., 2020), adaptions have been made in groups 5 and 7. All diets were purchased from Ssniff-Spezialdiäten GmbH (Soest, Germany) and stored at refrigerator temperature or, in case of polyunsaturated fatty acid (PUFA) containing diets, at −20°C in order to minimize oxidation (Jansen et al., 2013). Due to the long duration of the study, diets were obtained in consecutive batches, thus avoiding expiry. Each animal received tap water *ad libitum* and a daily amount of 4 g of diet. Moreover, each animal was weighed weekly and scored for body condition twice a week in order to monitor its nutritional status. Feeding the experimental diets started at the age of 6 weeks and proceeded for the entire course of the study, except for short regeneration periods due to poor body condition and animal loss in the B-vitamin deficiently fed groups (summarized in Supplementary Table 2). Sulfathiazole sodium was only supplemented in the vitamin-B deficient groups as was done in other studies (Saini et al., 2016; Nieraad et al., 2020, 2021a). A disadvantage is that sulfathiazole sodium itself cannot be controlled for in the present study, since we have not included it in the groups that received a normal control diet.

**TABLE 1:** Experimental groups.

Group number	Genotype	Diet	Abbreviation
1	C57BL/6J wild type	Control	C (WT)
2	*App^NL–G–F^* knock-in	Control	C (KI)
3	*App^NL–G–F^* knock-in	Vitamin B deficient	B-DEF
4	*App^NL–G–F^* knock-in	Vitamin B enriched	B-ENR
5	*App^NL–G–F^* knock-in	Vitamin B deficient and PUFA supplemented	B-DEF+PUFA-ENR
6	*App^NL–G–F^* knock-in	Vitamin B enriched and PUFA supplemented	B+PUFA-ENR
7	*App^NL–G–F^* knock-in	Vitamin B deficient and betaine supplemented	B-DEF+BET-ENR

### Sample Collection

**FIGURE 1:**
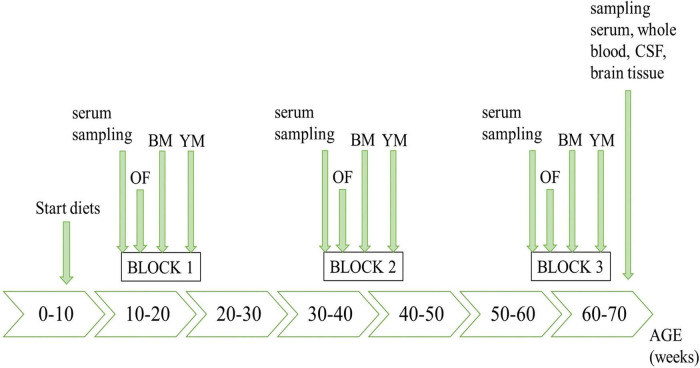
Timeline of the *in vivo* experiments. OF, open field test; BM, Barnes maze; YM, Y-maze.

The timeline of the *in vivo* study course is illustrated in Figure 1, highlighting all sampling steps as well as the schedule of behavioral testing.

In the first serum sampling steps, puncturing the facial vein with 5 mm Goldenrod animal lancets (MEDIpoint, Mineola, NY, United States) enabled blood collection (max. 170 μL per 25 g mouse, according to GV-SOLAS animal welfare guidelines). Further processing to serum is described below. At the end of the study, at the age of nearly 70 weeks, different biological matrices were sampled for subsequent analyses. For that purpose, the mice were deeply anaesthetized using 200 mg/kg (body weight) ketamine (Vétoquinol GmbH, Ismaning, Germany) and 10 mg/kg (body weight) xylazine (Bayer Health Care, Leverkusen, Germany). After the cessation of reflexes had been confirmed, we sampled cerebrospinal fluid (CSF) by cisterna magna puncture. Therefore, we shaved the back of the mice’ head and made a small incision. Tissues were gently removed as much as needed using extra fine graefe forceps (FST, Heidelberg, Germany) and pulled borosilicate glass capillaries (WPI, Friedberg, Germany) were utilized to carry out the puncture with the help of a surgical stereoscope (Zeiss, Oberkochen, Germany). Approximately 10 μL of clear CSF were obtained per mouse, frozen on dry ice and stored at −80°C for further use. Subsequently, blood was taken cardially and animals were killed by cervical dislocation and brains were harvested. The blood was transferred into tubes, containing either a clotting factor or EDTA (Sarstedt, Nümbrecht, Germany). In the serum tubes, blood was allowed to fully coagulate for 15–30 min before it was centrifuged (3,200 *g*; 4°C) for 15 min. Whole blood was immediately analyzed, whereas serum was frozen on dry ice and stored at −80°C for subsequent analyses. For the brains, a cross sectional part of about 100 mg tissue posterior to bregma was removed using razor blades and a brain matrix (WPI), weighed, frozen in liquid nitrogen, and stored at −80°C. Cross sections were taken independently of specific brain structures, as the *App^NL–G–F^* previously proved to exhibit amyloidosis throughout the whole brain (Sakakibara et al., 2019; Nieraad et al., 2020). The order of animals during sampling steps and euthanasia was defined according to a randomization list.

### Biochemical Analysis of Homocysteine and Homocysteic Acid

Quantification of HCys and HCA in serum samples using liquid chromatography in combination with tandem mass spectrometry (LC-MS/MS) was performed as described in detail in previous publications of our group (Gurke et al., 2019; Nieraad et al., 2020, 2021a).

### Behavioral Testing

In the present study, the animals’ cognitive performance was tested in three different settings and in three time blocks across the course of the study (15–21, 38–44, and 60–66 weeks of age). All outcomes in the open field test, Barnes maze and Y-maze were automatically detected by an overhead camera and analyzed with the corresponding EthoVision XT 13 software (Noldus, Wageningen, the Netherlands). Between the trials, testing systems were cleaned with 70% ethanol in order to remove odor cues. Every experiment was performed between 8 a.m. and 1 p.m. during the light phase and the animals were allowed to acclimatize to the experimental room 30 min before the start of behavioral testing. The order in which the animals were tested, was defined according to a randomization list.

#### Open Field

Habituation and anxiety-related behavior of the mice were evaluated using an open field apparatus (Hugo Sachs Elektronik, March-Hugstetten, Germany). Procedures were conducted as described recently (Nieraad et al., 2020), using the habituation ratio as an indicator for intrasession habituation (Equation 1). The position in the arena and the distance moved by the mice was continuously recorded and further analyzed for the total duration of 30 min and for different time bins.


(1) Habituationratio=activity(final 5min)actitvity(final 5min+initial 5min)

#### Barnes Maze

Procedural details for the acquisition phase and probe trial in the Barnes maze were previously reported in depth (Nieraad et al., 2020). Briefly, spatial learning and memory performance of the animals were assessed by measuring the time the mice needed to escape from an aversive, bright, and open space, while orienting to different visual cues positioned around the maze. The test consisted of two consecutive phases: in the acquisition phase, the mice needed to learn the position of an escape box (target) in 3 min sessions on four subsequent days of training, and escape latencies were recorded. In the probe trial on day 5, the mice were subjected to the maze after removal of the escape box for 1.5 min. The exact position of the target in the maze was randomly assigned per animal and it was ensured that when repeating the test in time blocks two and three, reversal settings were applied for each mouse. We chose the use of the Barnes maze (BM) instead of the Morris water maze (MWM), since it has been found be less stressful for animals (especially in mice), because there is no water immersion and the subsequent high corticosterone increase which may affect the performance of animals (Holmes et al., 2002; Harrison et al., 2009; Gawel et al., 2019). In addition, the BM is not so physically demanding for rodents than the MWM.

#### Y-Maze

As a testing system for working memory, we used a Y-maze apparatus (Hugo Sachs Elektronik) to further characterize cognitive abilities. For this purpose, each mouse was placed into the center of the maze and explored the three symmetrical arms for a period of 8 min under reduced light (30 lux). Sequence and number of arm entries were automatically recorded and used for the calculation of spontaneous alternation (Equation 2). Thus, an alternation indicates that sequential entry into all three different arms was completed (e.g., A, B, and C).


(2) $$\begin{array}{c} Spontaneous\;alternation\left(\%\right)\\ \;=\frac{numberofalternations}{\left(totalnumberofarmentries-2\right)}\times 100 \end{array}$$

### Hematology

Immediately after cardiac puncture, 10 μL whole blood from each mouse were measured in a Celltac α MEK-6500K automated hematology analyzer (Nihon Kohden, Rosbach v.d.H., Germany) in order to examine potential effects of AD-like genotype, HHCys or dietary interventions on various hematological parameters.

### Olink Proteomics

Protein analysis of 92 mouse biomarkers was performed using the Olink mouse exploratory panel (Olink, Uppsala, Sweden). A list comprising all proteins in this panel and their relation to diverse biological processes is provided by the supplier.^2^ We used both serum and CSF from the animals, from which volumes of 1 μL were required for the measurement. Due to limited capacity on the plate, animals of the groups 1, 2, 3, 4, and 6 (resp. *n* = 8; 11; 8; 9; 8) were included, whereas groups 5 and 7 were excluded from this analysis. The total number of observations in the proteome analysis was limited by animal loss or failure of appropriate sampling volumes. Plate loading proceeded randomly and the pipetter was blinded to the experimental groups at this stage of the experiment. All quality control parameters were applied according to the supplier’s instructions. The Olink proteomics technology is based on the proximity extension assay (Assarsson et al., 2014) and results in relative quantification of protein biomarkers in the Normalized Protein eXpression (NPX) unit, which is an arbitrary unit on log2 scale. Thereby, higher NPX values indicate higher protein concentrations. Resulting data were further analyzed for enrichment with the help of Gene-ontology (GO) and PANTHER classification system.^3^

### Quantification of Aβ

The amounts of cerebral Aβ peptides, i.e., the humanized and modified Aβ42 from the *App^NL–G–F^* KI mice, were determined in this study using ELISA (KHB3441, Invitrogen, ThermoFisher). The Aβ extraction protocol reported here is similar to that in other studies using *App^NL–G–F^* KI mice (Jacob et al., 2019; Rice et al., 2020). Initially, brain tissue sections of about 100 mg were thawed on ice in 1 mL of pre-cooled extraction buffer, which consisted of 5 M guanidine hydrochloride (GuHCl), 50 mM TRIS, and 1x protease inhibitor cocktail (Sigma-Aldrich), adjusted to pH = 8. With the help of GuHCl, not only the soluble but also the insoluble portion of Aβ could be determined. One-step mechanical lysis was done in a FastPrep-24 5G homogenizer (MP Biomedicals, Eschwege, Germany) using the mouse brain program (6 m/s; 40 s) and homogenates were shaken (300 rpm) afterward for 3.5 h at room temperature. After 1:10 dilution with cold PBS, including protease-inhibitor, the homogenates were centrifuged (20,000 *g*; 20 min; 4°C) and supernatants were further diluted 1:200 in standard diluent buffer. Loading GuHCl in this dilution did not pose the risk of disintegrating the ELISA plate. Plate loading and absorption measurement then proceeded according to the supplier manual. Peptide concentrations were finally derived from absorption data via four parameter logistic curve fit model, and under consideration of the dilution factor, were normalized to the initial weights of the tissue sections.

### Statistical Analyses

Initially, the number of mice per group had been estimated through statistical power calculation.^4^ SPSS Statistics 26 (IBM, Ehningen, Germany) was used for all statistical analyses in the present study. Prior to data analysis, extreme outliers (>3x interquartile range; summarized in Supplementary Table 2) were excluded, followed by normality testing using Shapiro Wilk test. As Gaussian distribution could not be assumed in most data sets, we mainly performed non-parametric Mann-Whitney-*U* tests for the pairwise comparisons (group 1 vs. 2 and group 2 vs. 3–7). A *p*-value (two-tailed exact sig.) lower than 0.05 was considered statistically significant. Furthermore, non-parametric (group-unspecific) Spearman rank correlation tests were applied. Data were expressed as median ± interquartile range, pooled for males and females, using GraphPad Prism 7 (San Diego, CA, United States). Merely for the cell viability assay, normality testing indicated Gaussian distribution and therefore, parametric analysis (*t*-test) was performed and results are depicted as means ± standard error of the mean.

## Results

### Cell Viability *In vitro* Assay

**FIGURE 2:**
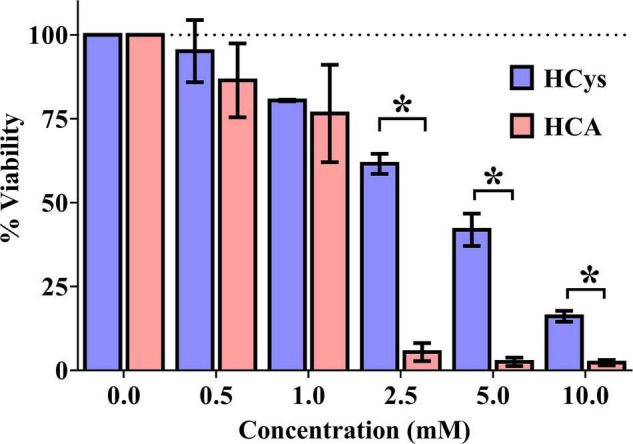
Luciferase-based cell viability measurement of primary cortical neurons (10,000 per well) after 24 h of incubation with homocysteine (HCys) or homocysteic acid (HCA); mean ± SEM (*n* = 3); *p* < 0.05 (*t*-test) considered statistically significant (*).

Luciferase-based testing showed a concentration-dependent effect of HCys and HCA on the viability of primary neurons (Figure 2). In relation to the vehicle control (100% viability), increasing concentrations of each compound were associated with increasing levels of cell death. At a measured concentration of at least 2.5 mM, the difference in neurotoxicity between HCys and HCA became significant (2.5 mM *p* < 0.001; 5.0 mM *p* = 0.011; 10.0 mM *p* = 0.005). Celecoxib (1 mM), as a positive control, decimated almost all cells in the respective wells (not shown).

### Biochemical Analysis of Homocysteine and Homocysteic Acid

**FIGURE 3:**
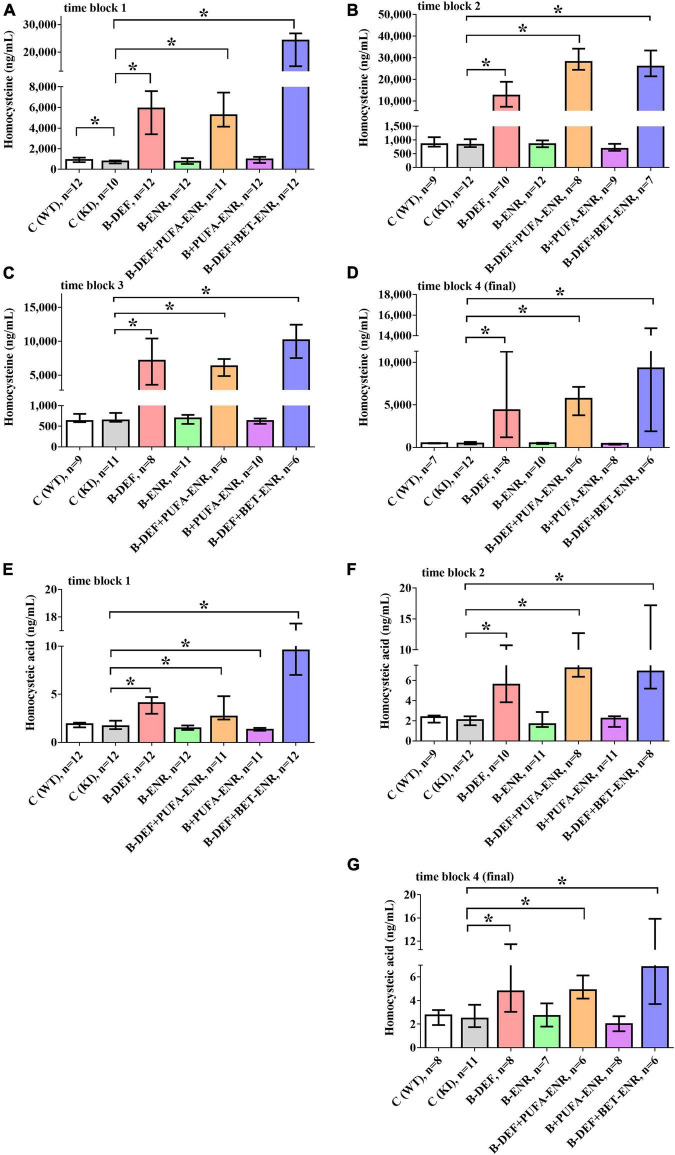
*In vivo* serum levels of homocysteine (HCys) and homocysteic acid (HCA); **(A)** HCys, time block 1; **(B)** HCys, time block 2; **(C)** HCys, time block 3; **(D)** HCys, final sampling step; **(E)** HCA, time block 1; **(F)** HCA, time block 2; **(G)** HCA, final sampling step; no data obtained for homocysteic acid in time block 3 due to sample loss while sample preparation; all samples analyzed via LC-MS/MS; median ± IQR; outliers beyond 3-fold IQR removed; *p* < 0.05 (Mann-Whitney-*U*-test) considered statistically significant (*).

*In vivo* serum levels of the amino acids HCys and HCA were analyzed several times longitudinally during the study course using LC-MS/MS analysis (Figure 3). Consequent to the dietary B-vitamin restriction, HCys levels were shown to be significantly elevated in time block 1 (B-DEF *p* < 0.001; B-DEF+PUFA-ENR *p* < 0.001; and B-DEF+BET-ENR *p* < 0.001), time block 2 (B-DEF *p* < 0.001; B-DEF+PUFA-ENR *p* < 0.001; and B-DEF+BET-ENR *p* < 0.001), time block 3 (B-DEF *p* < 0.001; B-DEF+PUFA-ENR *p* < 0.001; and B-DEF+BET-ENR *p* < 0.001), and at the end of the experiment (B-DEF *p* < 0.001; B-DEF+PUFA-ENR *p* < 0.001; and B-DEF+BET-ENR *p* < 0.001). A difference between WT and KI control mice just reached significance in time block 1 (*p* = 0.043) and was not further confirmed at any other sampling step. We did not detect a statistically significant impact of B-vitamin and/or PUFA supplementation on serum HCys compared to KI control mice.

Also, HCA serum levels were elevated in all three B-vitamin deficient groups at every sampling step (time block 1: B-DEF *p* < 0.001; B-DEF+PUFA-ENR *p* < 0.001; and B-DEF+BET-ENR *p* < 0.001; time block 2: B-DEF *p* < 0.001; B-DEF+PUFA-ENR *p* < 0.001; B-DEF+BET-ENR *p* < 0.001; and final sampling step: B-DEF *p* = 0.041; B-DEF+PUFA-ENR *p* = 0.001; and B-DEF+BET-ENR *p* = 0.005). For time block 3, no HCA data were obtained due to sample loss while sample preparation. No effect of the *App^NL–G–F^* KI genotype on HCA levels was detected. The diet supplemented with B-vitamins and PUFAs resulted in significantly different HCA compared to KI control, however, only in time block 1 (*p* = 0.026).

Overall, hyperhomocysteinemic mice supplemented with betaine tended to build up higher serum levels of both HCys and HCA than animals from the other hyperhomocysteinemic groups.

### Behavioral Testing

**FIGURE 4:**
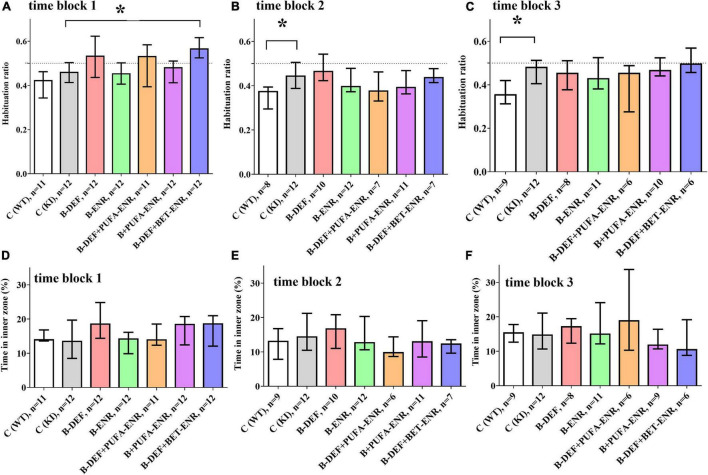
Open field testing in time block 1–3: **(A,C,E)** intrasession habituation (lower habituation ratio meaning a higher level of intrasession habituation) and **(B,D,F)** anxiety-related behavior; median ± IQR; outliers beyond 3-fold IQR removed; *p* < 0.05 (Mann-Whitney-*U*-test) considered statistically significant (*).

In the open field test, two different behavioral domains were investigated. Firstly, calculation of the habituation ratio, based on the distance moved by the mice in different time bins (Equation 1), enabled assessment of habituation learning, whereby a lower ratio is associated with a higher level of intrasession habituation. Figures 4A–C depicts habituation ratios for all three time blocks. In the first time block, hyperhomocysteinemic KI mice supplemented with betaine showed a lower level of intrasession habituation compared to KI control animals (*p* < 0.001). However, this effect did not prove to be consistent when taking the subsequent time points into consideration. A difference between WT and KI control mice became statistically significant in time block 2 (*p* = 0.012) and was confirmed in time block 3 (*p* = 0.007), also at a higher effect size *r*. Comorbid AD-like pathology and HHCys did not further impair habituation learning compared to KI control mice.

Secondly, the time spent in the inner zone of the arena was used as an indicator for anxiety-related behavior: the lower the time in the inner zone, the higher is the level of anxiety. For this parameter, no differences between the experimental groups were detected (Figures 4D–F).

Data derived from the Barnes maze and Y-maze did not reveal consistent differences between the experimental groups and can be found in the Supplementary Figures 1, 2 for the Barnes maze and Supplementary Figure 3 for the Y-maze (Supplementary Results).

### Hematology

**FIGURE 5:**
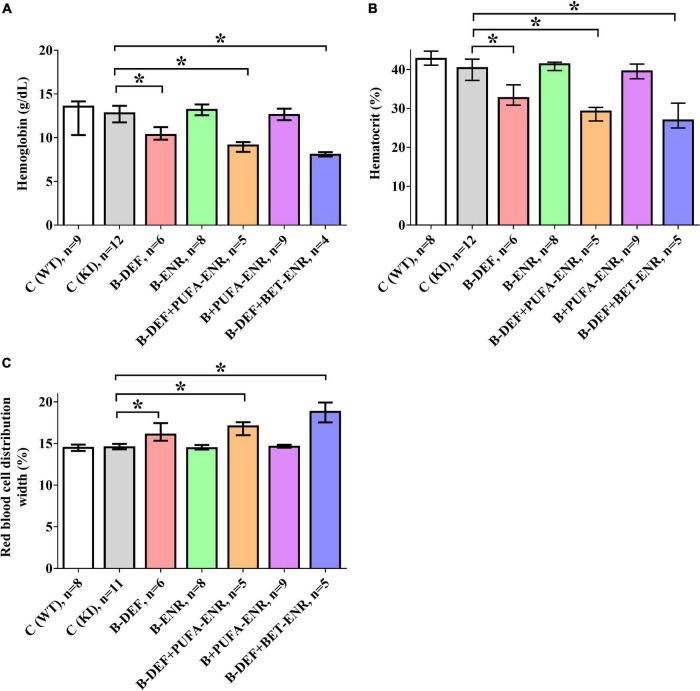
Hematological parameters in whole blood at the age of 67–68 weeks; median ± IQR; outliers beyond 3-fold IQR removed; *p* < 0.05 (Mann-Whitney-*U*-test) considered statistically significant (*); **(A)** hemoglobin concentration; **(B)**: hematocrit; **(C)** red blood cell distribution width coefficient of variation.

For the evaluation of several hematological parameters, whole blood from the mice was taken and measured at the final sampling step. As illustrated in Figure 5, we detected significant group differences in parameters related to red blood cells. In all three hyperhomocysteinemic groups (group 3; 5; 7), decreased hemoglobin levels (B-DEF *p* = 0.039; B-DEF+PUFA-ENR *p* = 0.018; and B-DEF+BET-ENR *p* = 0.013) were measured, as well as reduced hematocrit (B-DEF *p* = 0.007; B-DEF+PUFA-ENR *p* = 0.002; and B-DEF+BET-ENR *p* = 0.001). Furthermore, a higher red cell distribution width, meaning an increased range between smaller and larger erythrocytes, was detected in these groups (B-DEF *p* = 0.002; B-DEF+PUFA-ENR *p* < 0.001; and B-DEF+BET-ENR *p* < 0.001).

As indicated by non-parametric Spearman rank correlation test, HCys negatively correlated with hemoglobin (rs = −0.369; *p* = 0.006) and hematocrit (rs = −0.388; *p* = 0.004) and positively correlated with red blood cell distribution width (rs = 0.340; *p* = 0.012).

### Olink Proteomics

Proteome analysis revealed significant up- or down-regulation of various protein biomarkers in serum and CSF. Only data from groups 1–3 are depicted here due to the absence of effects of dietary supplementation with B-vitamins and/or PUFA. These merely had a low effect-size impact on a few markers, did not result in findings in both serum and CSF and, finally, did not reveal any enrichment for particular biological processes at all in the GO-analysis. Consequently, in this section we focus on effects of the *App^NL–G–F^*-related AD-like pathology on the one hand and additionally induced HHCys on the other hand.

Details on the deregulation of all affected proteins, as well as effect sizes and other parameters are summarized in Table 2. Compared to WT, we detected 14 proteins, for which concentrations differed significantly in KI mice. Especially differences in CSF markers revealed large effect-sizes for this group comparison. B-vitamin deficiency and concomitant elevation of HCys affected 22 different proteins, mainly in serum of the animals. Hyperhomocysteinemic mice expressed merely few differences in CSF in comparison to KI mice fed with control chow.

**TABLE 2:** Impact of the AD-like genotype and HHCys on proteins that were found to be significantly up- or downregulated in the Olink proteomics analysis; effect size was calculated as |*r*| = *z*/√*N*.

*App^NL–G–F^* knock-in genotype (group 1 vs. 2)						Hyperhomocysteinemia (group 2 vs. 3)					
Protein		Matrix	*N*	*P* value	Effect size *r*	Protein		Matrix	*N*	*P* value	Effect size *r*
CCL3	↑	Serum	19	0.016	0.549	CCL2	↓	Serum	19	<0.001	0.777
CLMP	↑	Serum	19	0.041	0.474	CXCL9	↓	Serum	19	0.007	0.606
ERBB4	↑	Serum	19	0.007	0.606	DLL1	↓	Serum	18	0.012	0.586
GFRA1	↑	Serum	19	0.033	0.493	EDA2R	↓	Serum	19	0.016	0.549
IGSF3	↓	Serum	8	0.036	0.791	EPCAM	↑	Serum	14	0.038	0.563
NOTCH3	↑	Serum	19	0.026	0.511	FAS	↓	Serum	19	0.007	0.606
VEGFD	↑	Serum	19	0.033	0.493	FSTL3	↓	Serum	19	0.026	0.511
CCL3	↑	CSF	19	<0.001	0.833	GFRA1	↓	Serum	19	<0.001	0.833
CNTN1	↑	CSF	19	0.041	0.474	IGSF3	↑	Serum	9	0.032	0.735
ENO2	↑	CSF	19	<0.001	0.833	IL1a	↑	Serum	19	0.026	0.511
HGF	↑	CSF	18	<0.001	0.838	IL23R	↓	Serum	19	0.001	0.701
RGMA	↓	CSF	17	0.002	0.710	LGMN	↓	Serum	19	0.007	0.606
TNR	↑	CSF	18	<0.001	0.800	MATN2	↓	Serum	18	0.034	0.503
TPP1	↑	CSF	19	0.005	0.625	S100A4	↓	Serum	19	0.007	0.606
						TGFBR3	↓	Serum	19	0.003	0.663
						TNFRSF11B	↓	Serum	19	0.001	0.720
						TNFRSF12A	↓	Serum	19	0.003	0.663
						TPP1	↓	Serum	19	0.001	0.701
						VSIG2	↓	Serum	17	0.025	0.544
						WISP1	↓	Serum	19	0.012	0.568
						ACVRL1	↓	CSF	13	0.035	0.594
						DLK1	↓	CSF	13	0.030	0.609

**FIGURE 6:**
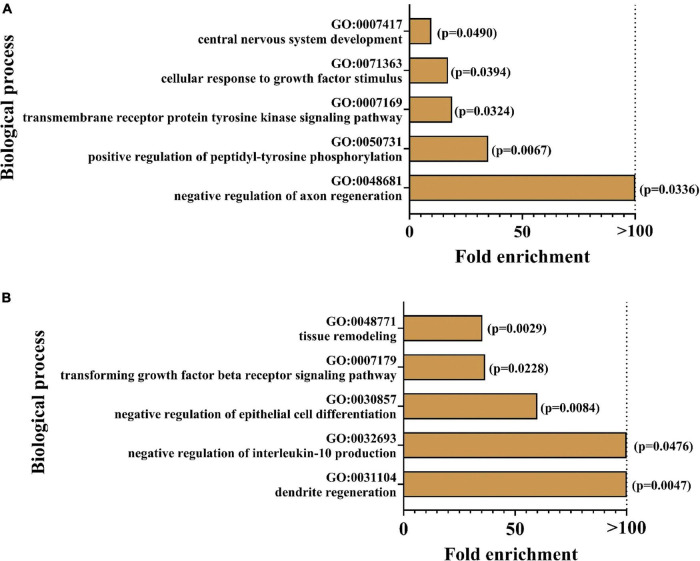
Enrichment analysis was performed using geneontology.org (GO); top five of the most strongly enriched biological processes are depicted for **(A)** *App^NL–G–F^* knock-in versus C57BL/6J wild type mice and **(B)** hyperhomocyste-inemic versus control *App^NL–G–F^* knock-in mice; *p*-values are expressed in brackets for each enriched biological process.

In order to determine biological processes for which the markers were enriched, GO analysis was conducted. Figure 6 illustrates the top 5 enriched pathways for the focussed group comparisons. For both cases, especially pathways in the neuronal context seemed to be affected: the strongest enrichments were found for the regulation of axon regeneration in *App^NL–G–F^* control mice and dendrite regeneration in hyperhomocysteinemic *App^NL–G–F^* mice, respectively. As the presented GO analysis has to be recognized purely as an association analysis, the assessment of the single deregulated markers is mandatory and the respective biological matrix (serum, CSF) must be taken into consideration. An in-depth discussion of all affected proteins is provided subsequently (see section “Discussion − Olink Proteomics”).

**FIGURE 7:**
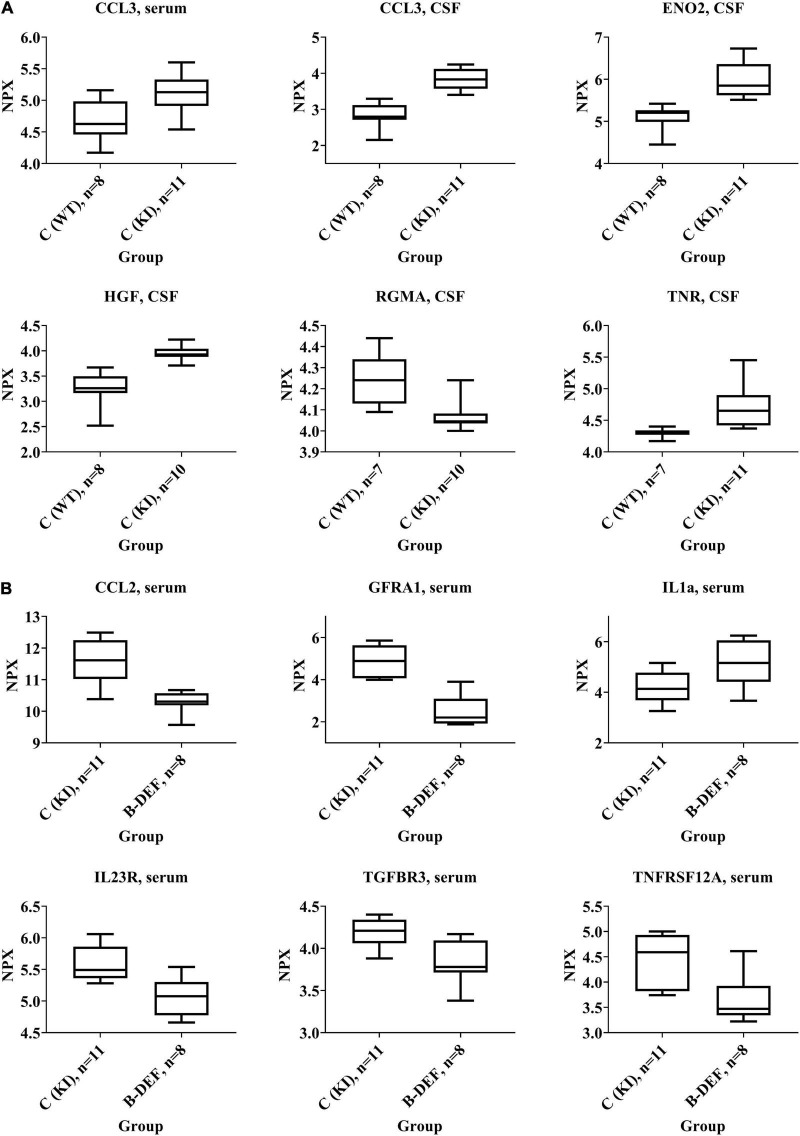
Selected protein biomarkers that were differentially regulated in **(A)** *App^NL–G–F^* knock-in versus C57BL/6J wild type mice and **(B)** hyperhomocysteinemic versus control *App^NL–G–F^* knock-in mice; age 67–68 weeks; differences in the protein quantities depicted here did all reach statistical significance (*p*-values < 0.05); normalized protein expression (NPX) is an arbitrary unit on log2-scale; high NPX means high protein concentration.

Some selected protein markers are depicted separately and highlighted in Figure 7, mainly because of large effect-sizes (cf. Table 2). Protein markers referring to neuronal function or (neuro-) inflammation were the focus of this study and proved to be deregulated especially in CSF of KI control mice (e.g., ENO2, HGF, RGMA, and TNR). With respect to a hyperhomocysteinemic impact, Figure 7B equally highlights neuron and inflammation-related parameters – however, mostly in serum − but also parameters of relevance apart from the neurodegenerative context (e.g., TGFBR3), as discussed further below (see section “Discussion − Olink Proteomics”). Despite apparently strong effect sizes, we did not separately depict and further discuss IGSF3 due to a decreased number of observations and therefore, a higher level of uncertainty in the proteome analysis.

### Quantification of Aβ

**FIGURE 8:**
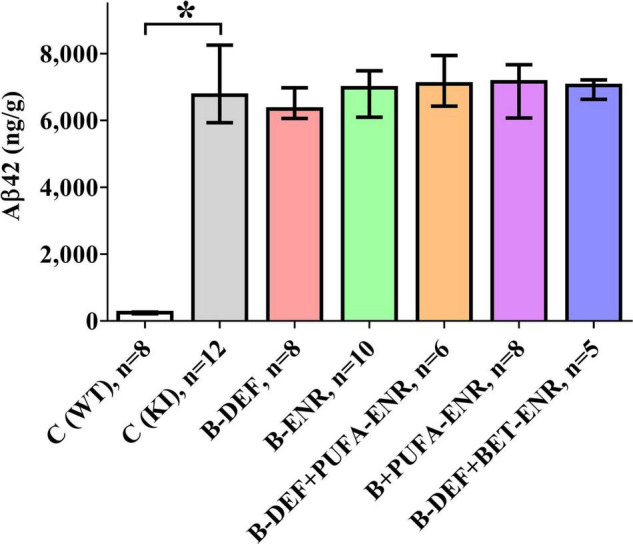
Total Aβ42 level (GuHCl-soluble) per gram wet brain tissue in a 100 mg cross-section; age 67–68 weeks; median ± IQR; outliers beyond 3-fold IQR removed; *p* < 0.05 (Mann-Whitney-*U*-test) considered statistically significant (*).

Analysis of brain section homogenates using ELISA revealed cerebral Aβ42 levels that are depicted in Figure 8. The difference in Aβ levels between the different genotypes was highly significant (*p* < 0.001), whereas none of the other groups showed a statistically significant difference in comparison to KI control mice.

Correlation analysis revealed positive correlation between cerebral Aβ42 and CSF protein markers, e.g., CCL3 (rs = 0.476; *p* = 0.001), ENO2 (rs = 0.505; *p* = 0.001), HGF (rs = 0.507; *p* = 0.001), and TNR (rs = 0.491; *p* = 0.001).

## Discussion

The primary goal of this study was to examine the effects of long-term B-vitamin deficiency or dietary supplementation with specific micronutrients on cognitive performance in the *App^NL–G–F^* KI mouse model for AD. We were particularly interested in elucidating a potential aggravation of AD-like pathology by the amino acids HCys and HCA. Additionally to the phenotypic characterization of the mice using different behavioral tests, we performed analyses of serum HCys/HCA, hematological parameters and cerebral Aβ burden, as well as serum and CSF proteomics. A second aim was to further characterize the *App^NL–G–F^* model for AD. The results of the presented behavioral tests, as well as the assessment of plaque burden, mostly confirmed the findings of our previous *in vivo* study at a higher age of the animals in this long-term experiment. However, previous findings have been extended especially by the *ex vivo* experiments consisting of hematological and proteome analyses, but also by revelations through group and diet adaptions, as well as the findings of the *in vitro* assay performed in the presented study. These aspects are subsequently discussed in more detail.

### Homocysteine and Homocysteic Acid

The *in vitro* results of the present study confirmed the hypothesis that HCys is actually less neurotoxic than its oxidative metabolite HCA. In both cases, marked cytotoxicity was detected at concentrations beyond 1 mM, which is higher than *in vivo* relevant levels of these compounds. However, these values in isolated cells might not be directly translatable to animal or human studies, but have to be discussed in the context of the setting of the *in vitro* assay. Firstly, despite using isolated primary rat cortical neurons that are biologically more relevant than artificial cell lines, it has to be emphasized that other types of neurons such as hippocampal neurons might have been more vulnerable to HCys and HCA than cortical neurons that were used in the present study (Lockhart et al., 2000; Loureiro et al., 2008). Secondly, (higher) neurotoxicity of HCys and HCA may synergistically derive from a combination with hallmarks of AD, e.g., amyloid pathology (Hasegawa et al., 2005), and vice versa, also HCys and HCA can potentially deteriorate toxicity of other AD hallmarks (Li et al., 2014). Thirdly, incubation of cells with the compounds occurred for merely 24 h in this short-term assay and is, therefore, much shorter than chronic exposure *in vivo*. Moreover, neurotoxicity exerted by the NMDA receptor is dependent on the height of glycine levels, due to different HCys-binding sites at the NMDA receptor: higher glycine levels in the brain are suggested to correlate with higher HCys-driven NMDA agonism and consequently higher toxicity occurs already at lower HCys concentrations (Lipton et al., 1997; Moretti and Caruso, 2019). In accordance with our results, neurotoxicity of HCys has been reported >1 mM, whereas HCA is supposed to be more potent (Vladychenskaya et al., 2006). We consequently also determined the level of HCA in addition to HCys in serum in the animals in the present *in vivo* study.

As revealed by LC-MS/MS analysis, HHCys was successfully induced in *App^NL–G–F^* mice by feeding a B-vitamin restricted diet (group 3), which was additionally supplemented with either PUFA (group 5) or betaine (group 7). The absence of vitamin B12 and folate is suggested to result in a disrupted remethylation cycle and chronically elevated serum HCys; additionally enhanced by the lack of vitamin B6, which an essential cofactor in the transsulfuration pathway of HCys (Nieraad et al., 2021b). As recently reviewed, one carbon metabolism disturbance, especially decreased S-adenosylmethionine and increased S-adenosylhomocysteine and concomitantly reduced methylation capacity, affects numerous metabolic pathways (Nieraad et al., 2021b). Epigenetic regulation, as a consequence of diminished supply with methylation groups, might also explain our findings in the present proteome analysis, i.e., the deregulation of several protein biomarkers.

Dayal and Lentz hypothesized that long-term dietary treatment might be associated with secondary metabolic effects, leading to varying HCys levels between different sampling points throughout the study course (Dayal and Lentz, 2008). In our study, variability between the time blocks might, to some extent, also result from an absent randomization of samples over the different LC-MS/MS runs during the analysis procedure. This was not possible, because of long-term sample storage duration, in this case due to the long-term study design. However, significant HHCys (groups 3; 5; 7) was clearly confirmed prior to each block of cognitive testing. In the current study, we were more interested in the comparison between different experimental groups at each particular time point than in longitudinal comparisons. No consistent impact on the height of HCys and HCA levels was detected, neither for the *App^NL–G–F^* -related amyloid pathology nor for B-vitamin treatment. In the case of PUFA, researchers reported findings pro (Huang et al., 2018) and contra (Martínez-Vega et al., 2015) beneficial effects on HCys levels. This effect proved to be absent in the present mouse study, as revealed by group 5 (hyperhomocysteinemic + PUFA-supplemented), which had been modified compared to our previous *in vivo* study (Nieraad et al., 2020). With group 7 (hyperhomocysteinemic + betaine-supplemented), another adaption was implemented in the current study: we attempted to mitigate HHCys in a B-vitamin-independent manner in order to differentiate between potentially detrimental effects of HCys itself and the underlying B-vitamin deficiency. However, this question could not be answered, since serum HCys was not normalized by the betaine-supplemented diet, but elevated compared to *App^NL–G–F^* control animals. Interestingly, it has also been reported previously that betaine did not lead to a decrease in HCys or even further increased levels (Kunisawa et al., 2015; Ahmad et al., 2019). A reason for this might be reduced food intake by the mice due to a potential aversion against the betaine-containing chow and, as a consequence, even lower intake of dietary choline and additionally decreased methylation capacity. Overall, pathologically elevated levels of serum HCys and HCA severely affected the general health condition and resulted in decreased body weight, skin problems and a higher death rate in the respective groups of mice.

In hyperhomocysteinemic mice, erythrocyte-related parameters proved to be affected, as revealed by whole blood analysis. However, the question remains whether these findings resulted from elevated HCys or from the underlying deficiency in vitamin B6, B12, and folate, a lack of which is known to be responsible for the onset of anemia. Nevertheless, there are also hints that elevated levels of HCys itself might modulate severity of the reported effects. Mice of group 7 (hyperhomocysteinemic + betaine-supplemented), which showed the highest serum HCys of all three hyperhomocysteinemic groups (Figure 3), also expressed the most distinct effects on red blood cell-related parameters (Figure 5), although B-vitamin deficiency was not different for all three groups. Correlation analysis indicated a (weak) negative correlation between HCys and hemoglobin and hematocrit, as well as a positive correlation between HCys and red blood cell distribution width (RDW) according to a human study (Peng and Pan, 2017). This potential contribution of HCys to vascular aspects of cognitive impairment is further addressed below in the proteomics section (see section “Discussion − Olink Proteomics”). The induced anemia and the consequent reduced supply of oxygen likely explains the poorer general condition of the hyperhomocysteinemic mice compared to control animals.

### Aβ Pathology and Behavioral Outcome

The results of the cerebral Aβ42 analysis indicated successful induction of the AD-like amyloid pathology in the *App^NL–G–F^* model, which furthermore, is in accordance with the immunohistochemical data of our former *in vivo* study (Nieraad et al., 2020). Also in agreement with our previous study (Nieraad et al., 2020), cerebral amyloidosis was not affected by different dietary micronutrients or by elevated serum HCys/HCA due to vitamin-B deficiency. In contrast to our previous study, here, we measured both the insoluble and soluble portion of Aβ42, which is overexpressed in the *App^NL–G–F^* mouse model (Saito et al., 2014) and plays a major role in amyloid pathology (Grimm et al., 2017). Brain tissue levels of Aβ42 lay within ranges that have been reported for *App^NL–G–F^* in similar investigations (Saito et al., 2014; Jacob et al., 2019), however, absolute values might not be exactly comparable with other trials, as different ELISA systems and different antibodies have been used. Also, antibodies may exhibit varying binding affinity in the case of the WT control group compared to the KI mice, which contained an altered Aβ sequence due to humanization and insertion of the Arctic mutation. Nevertheless, in the current study, an absolute quantification of Aβ was not required and the method adequately enabled semi-quantification of Aβ, i.e., a comparison between the different groups with the *App^NL–G–F^* control mice.

Despite massive cerebral amyloidosis, only one of three behavioral tests revealed a significant impact of the AβPP-based KI mouse model. This further fuels the controversial discussion about the role of Aβ in neurodegeneration, which is frequently debated (Polis and Samson, 2019). It also underscores the more balanced approach and the consideration of other pathological mechanisms besides the Aβ pathology, as discussed by Banik et al. (2015), since AD is a multifactorial disease. The combination of disturbed Aβ metabolism with other typical hallmarks of AD, e.g., the characteristic Tau pathology, might model the complex pathology more comprehensively, as it has been attempted in older transgenic models such as the 3xTg-AD mouse model. However, it might be valuable to combine the versatility of such a model with the afore-mentioned advantages of the knock-in strategy in terms of model development.

The assessment of working memory performance in the Y-maze (Prieur and Jadavji, 2019), was implemented in the current study in order to enable comparability with previous investigations, which indicated ambiguous findings in this test (Saito et al., 2014; Whyte et al., 2018; Izumi et al., 2020; Narukawa et al., 2020; Uruno et al., 2020; Degawa et al., 2021). The apparent absence of an effect of the AD-like genotype in our KI mice might, however, be the result of the sub-optimal performance of the WT control mice in this test (approx. 55% spontaneous alternation versus approx. 70% in similar studies). In keeping with previous examinations, negative findings were obtained for spatial learning in the Barnes maze (Sakakibara et al., 2018; Hongo et al., 2020), whereas the open field test indicated *App^NL–G–F^* -related deterioration of habituation learning (cf. Nieraad et al., 2020). Test dependency of genotype effects of *App^NL–G–F^* in behavioral tests has recently been emphasized (Kundu et al., 2021). Meanwhile, the originators of the model have developed a 3rd generation AD mouse model, attempting to increase plaque pathology and neuroinflammation (Sato et al., 2021).

Similar to our former *in vivo* study (Nieraad et al., 2020), long-term HHCys did not exhibit exacerbation of cognitive performance, despite of significantly elevated levels of HCys and HCA over the major part of the lives of the mice. Therefore, our data do not reinforce the “homocysteine hypothesis” – at least in the present mouse model for the familial AD. It has to be emphasized that most AD models, comprising also the *App*^NL–G–F^** model, simulate the early-onset (familial) form of the disease and the very prominent and well-described hallmarks such as Aβ plaques and neurofibrillary tangles, whereas the more prevalent late-onset (sporadic) AD and the less-extensively investigated characteristics of the multi-facetted AD pathology might actually be the more valuable approach (Polis and Samson, 2019). Polis and Samson entitled the late-onset form a “brain expression of a systemic complex metabolic disorder.”

Micronutritional interventions comprising dietary supplementation with B-vitamins (B6, B12, and folate), PUFA, or betaine did not consistently improve learning and memory in the respective KI mice. The absence of B-vitamin effects is in accordance with a recent human trial (Kwok et al., 2020). Previous clinical and preclinical hints on potentially beneficial effects of betaine on cognition were, however, not confirmed in the disease model that we used (Sun et al., 2017; Ibi et al., 2019). As concluded by Zhao et al. (2018) findings on the role of betaine remain contradictory. With respect to effects of PUFA, our data (groups 5 and 6) are in accordance with previous publications that indicated negative results (Oksman et al., 2006; Arendash et al., 2007; Quinn et al., 2010) or laid emphasis on the limited or ambiguous level of evidence (Bos et al., 2016). In general, evidence on this topic is conflicting and equivocal, as elucidated previously (Van Dam and Van Gool, 2009; Ford and Almeida, 2019; Tinelli et al., 2019; Behrens et al., 2020; Wang et al., 2021). Recently, we summarized available human and animal literature on C1 metabolic disturbances, especially the metabolic role and pathomechanisms of HHCys, in the context of cognitive decline (Nieraad et al., 2021b).

As a limitation of the current study, significant but inconsistent dietary effects in behavioral tests, which are typically associated with variability to some extent, might in part be explained by decreased statistical power due to the death of several animals as a consequence of the long study duration and severe dietary restrictions.

### Olink Proteomics

These formatting of the 92 protein biomarkers in the mouse exploratory kit, many proved to be significantly affected by either AD-like pathology or comorbid amyloidosis with HHCys. In terms of enrichment analysis, it has to be emphasized that no “weighting” of the involved markers occurred at this stage, meaning that all proteins were included and treated equally in the GO analysis, independent of effect size or sampling matrix. As the GO analysis is purely an association analysis, the following section discusses effects of the individual deregulated protein markers on related biological processes.

Analysis of CSF, which is a relevant matrix in terms of neurodegenerative processes, mainly revealed effects that were related to the *App^NL–G–F^* genotype rather than to HHCys and, therefore, indicated an impact of the AβPP-based AD mouse model. This is in accordance with our findings in the open field test, where KI mice showed impaired habituation learning. Particularly neuronal markers were deregulated with large effect-sizes, such as HGF, ENO2, or TNR. The upregulation of HGF, for instance, is translationally relevant, since HGF has also been described to be elevated in AD patients (Tsuboi et al., 2003; Zhu et al., 2018). It functions as a nerve repair-promoting growth factor (Ko et al., 2018) and is therefore, most probably, upregulated as a consequence of neuronal damage due to cerebral amyloidosis in AD patients or in the animal model. Similarly, the neurotrophic factor ENO2, a marker for neuronal damage, has been related to neuroinflammation and neurodegeneration (Dichev et al., 2020). The function of TNR, another neuronal marker that proved to be upregulated in *App^NL–G–F^* mice, is strongly dependent on interacting molecules (Probstmeier et al., 2000). Particularly in combination with CNTN1, which was also elevated in CSF, it is known to inhibit neurite outgrowth (Pesheva et al., 1993; Apostolova et al., 2006). Complex and opposite effects on regulation of neuronal survival have been pro-posed to be exerted by RGMA, as reviewed recently (Tang et al., 2021). The downregulation of RGMA might be recognized as an attempt to repair the induced brain damage (Mothe et al., 2017), as described above for HGF and ENO2. Deregulation of other markers at a lower effect-size was found in the serum proteome. Hence, markers (ERBB4, NOTCH3, GFRA1, and VEGFD) that have also been associated with neuronal maintenance (Golden et al., 1999; Murphy et al., 2002; Mason et al., 2005), are probably of less relevance than the aforementioned CSF markers, at least in this mouse model. However, particularly VEGFD has previously been described as an inflammatory serum biomarker that correlates with AD (Popp et al., 2017). Furthermore, upregulation of the pro-inflammatory chemokine CCL3 was detected in both CSF and serum of *App^NL–G–F^* compared to WT mice. CCL3 is said to exert neuronal damage and deleterious effects on synaptic plasticity and learning performance by contributing to CNS inflammation by promoting migration of T-lymphocytes into the brain (Marciniak et al., 2015; Zenaro and Constantin, 2017; Martin and Delarasse, 2018). The induction of (neuro-) inflammatory processes is one hallmark of the AD-like pathology in *App^NL–G–F^* KI mice, as summarized by the developers of the model (Saito and Saido, 2018). Interestingly, we also found a positive correlation between cerebral amyloidosis and CSF markers such as CCL3, ENO2, HGF, and TNR.

On the other hand, the absence of findings in CSF of hyperhomocysteinemic KI mice – especially of pronounced changes in neuronal parameters – might explain the lack of cognitive aggravation in all three behavioral tests. Elevated HCys, however, proved to deregulate various proteins in serum of the animals. Serum levels of different markers related to neuronal functionality were downregulated, such as GFRA1, MATN2 or the two members of the tumor necrosis factor receptor superfamily TNFRSF12A and FAS (Desbarats et al., 2003; Sarabi et al., 2003; Tanabe et al., 2003; Korpos et al., 2015). In contrast, a decreased serum concentration of LGMN was detected with the opposite result compared to the above proteins, i.e., deletion of LGMN is associated with the alleviation of synapse loss (Zhang et al., 2014). However, a recent human trial did not confirm previous reports, according to which LGMN was a driver of the process of AD development (Zhang et al., 2020). Interestingly, the present proteome analysis revealed that (neuro-) inflammatory pathways were affected by HHCys in both directions: aside from the elevation of pro-inflammatory IL1a serum level (Dinarello, 2018), concentrations of other protein markers were decreased in comparison to KI control mice (IL23R, CCL2, CXCL9, S100A4, WISP1, and EDA2R), indicating also anti-inflammatory effects (Teng et al., 2015; Verhelst et al., 2015; Martin and Delarasse, 2018; Ambartsumian et al., 2019; Chen and Li, 2020). In other studies using different animal models, researchers found HCys-related elevations of mainly pro-inflammatory markers (Scherer et al., 2014; Braun et al., 2020; Elsherbiny et al., 2020). Rarely, anti-inflammatory effects or no effects on inflammation parameters at all have also been reported for HHCys (Durga et al., 2005; Pirchl et al., 2012). It is obvious that the studies are not completely comparable, since HHCys-induction varies between genetic, parenteral, and dietary methods, as well as between different durations of assessment. In terms of neuroinflammation, glial cells, particularly microglia, play an important role as key regulators (Saito and Saido, 2018). Prominent glia-related proteins such as TREM2 or ApoE that are frequently discussed in the context of AD pathology, were not part of the current proteome analysis, which therefore is a limitation of this study. Here, we cannot comprehensively review the complex interactions and all functions of the single inflammation markers in-depth. However, in sum, the absence of phenotypic impairment of the outcome of the behavioral tests might be explained by a potential abrogation of the inflammation-related effects, which are in part mutually counteractive and might also derive from an adaptive response of the organism to restore homeostasis.

As another HHCys-related effect, reduction in angiogenesis results from the downregulation of pro-angiogenic factors in serum (FAS, S100A4, TNFRSF12A, and TGFBR3; Wiley et al., 2001; Lambert et al., 2003; Ambartsumian et al., 2019; Wang et al., 2019) and CSF (DLK1 and ACVRL1; Urness et al., 2000; Huang et al., 2018), which might display an interesting mechanistic aspect in terms of vascular contribution to cognitive decline and dementia. HCys is known to exhibit a vascular impact, i.e., multiple deleterious effects on endothelial cells (Nieraad et al., 2021b). Endothelial dysfunction is, in turn, considered a contributor to oxidative stress in the brain, chronic cerebral hypoperfusion and AD development (Polis and Samson, 2019). As accordingly suggested in a recent meta-analysis, HCys plays a more important role in vascular dementia than in “pure” AD (Wang et al., 2021). In consideration of our hematological data, effects are probably aggravated as a result of the combination with reduced hemoglobin in the hyperhomocysteinemic mice, due to which neuronal exposure to oxygen is additionally diminished. Although it was apparently not sufficient to trigger phenotypic impairment in this study, it has previously been hypothesized that decreased capillary density in the brain and, therefore, decreased cerebral blood flow and supply of oxygen and glucose, contributes to the impairment of synaptic function and cognitive performance in elderly people as well as AD patients (Ambrose, 2014; Katsimpardi et al., 2014). Ambrose considered this approach underrated and it should be regarded, not in contrast to, but in addition to the amyloid hypothesis.

Apart from the actual research question of the current analysis, the detected combination of deregulated biomarkers also indicates other effects of HHCys. For instance, downregulation of TNFRSF11B, TGFBR3, FSTL3, and WISP1 and upregulation of IL1a in serum are associated with diminished bone mineralization, decreased bone anabolism, or increased bone catabolism (Lee et al., 2002; Hill et al., 2015; Maeda et al., 2015; Nam et al., 2015; Rochette et al., 2019). Accordingly, HCys has previously been suggested to be involved in osteoporosis-like pathology in patients, as reviewed earlier (Azzini et al., 2020). A potentially lighter skeletal structure, besides reducing appetite due to B-vitamin deficiency, is in accordance with the lower weights of the hyperhomocysteinemic mice in our study.

For both group comparisons, proteome analysis revealed deregulation of some neuron-related markers such as TPP1, ERBB4, NOTCH3, ENO2, RGMA, GFRA1, or DLL1, which are associated with developmental processes (Kaiser et al., 1989; Golden et al., 1999; Golding et al., 2000; Mason et al., 2005; Matsunaga, 2006; Rocha et al., 2009; Dimitrova et al., 2011). As the mice were already about 70 weeks old when we performed the analysis, neuronal developmental processes most probably did not play an important role any more, although neurogenesis proceeds to some extent in specific brain regions also in adult individuals (Apple et al., 2017). For that reason, other functions of these markers should probably be considered more relevant in older mice. In the case of the remaining deregulated markers, neither initial literature research using the uniprot database^5^ (Bateman et al., 2021) nor enrichment analysis indicated a direct connection with neuronal maintenance function or (neuro-) inflammation at all (EPCAM, VSIG2, FST, and CLMP).

A limitation of the present study setting relates to the HCys-elevation method we applied, meaning that the extent to which the observed effects are potentially biased by the dietary B-vitamin deficiency cannot be conclusively determined. Nevertheless, an advantage with the explorative setup of the Olink mouse panel is to highlight topics that were originally not the focus of the study and may open up new research ideas.

## Conclusion

In this study, the induction of *in vivo* disease models for AD-like pathology and HHCys have revealed findings that confirm and extend the results of our previous animal study. The amyloid-based *App^NL–G–F^* KI model proved to be associated with subtle changes in the present cognitive assessment, despite massive amyloidosis in the brain. This further challenges the role of Aβ plaque deposition as the sole or main culprit for AD, according to the frequently discussed amyloid hypothesis. A combination with additional characteristic AD hallmarks, such as Tau pathology, might also be a valuable approach in the context of knock-in mouse model development. Placing our findings in the light of the literature on HHCys, which is heterogeneous and in parts equivocal, it must be emphasized that elevated serum HCys and HCA did not cause aggravation of the induced amyloid pathology in this mouse model of familial AD. Although HHCys was not associated with impaired cognitive performance, a significant impact on erythrocyte-related parameters and angiogenesis was found, potentially indicating a mainly vascular contribution of HCys to cognitive decline by impairing the supply of neurons with oxygen and nutrients. Dietary supplementation with different micronutrients did not reveal improvement compared to *App^NL–G–F^* control mice in terms of Aβ burden or behavioral outcome and therefore, did not indicate prevention of cognitive decline in this AD mouse model. Our data on the explorative Olink mouse panel further characterize the *App^NL–G–F^* model and clarify to some extent the role of HCys in diverse biological processes. Therefore, proteome analysis data might present a springboard for subsequent investigations and should be validated in other preclinical models and/or human trials.

## Data Availability Statement

The original contributions presented in the study are included in the article/Supplementary Material, further inquiries can be directed to the corresponding author/s.

## Ethics Statement

The animal study was reviewed and approved by the local Ethics Committee for Animal Research in Darmstadt, Germany.

## Author Contributions

GG, NB, HN, and UT: conceptualization. RG, HN, NP, and ER: methodology. RG, HN, and A-KS: software. OA, HN, and RG: validation. HN: formal analysis. SL, A-KS, OA, NP, ST, YS, and HN: investigation. MH, NB, and GG: resources. NB, MH, and HN: data curation. HN: writing—original draft preparation. MP, UT, GG, HN, RG, YS, ST, A-KS, SL, ER, NP, MH, OA, and NB: writing—review and editing. HN: visualization. NB and MP: supervision. GG and MP: project administration. GG: funding acquisition. All authors have read and agreed to the published version of the manuscript.

## Conflict of Interest

The authors declare that the research was conducted in the absence of any commercial or financial relationships that could be construed as a potential conflict of interest.

## Publisher’s Note

All claims expressed in this article are solely those of the authors and do not necessarily represent those of their affiliated organizations, or those of the publisher, the editors and the reviewers. Any product that may be evaluated in this article, or claim that may be made by its manufacturer, is not guaranteed or endorsed by the publisher.

## Footnotes

1
https://www.random.org/

2
https://www.olink.com/products/target/mouse-exploratory/

3
http://geneontology.org/

4
http://www.biomath.info/power/

5
https://www.uniprot.org/
