# Supplementary material for: The Roles of Long-Term Hyperhomocysteinemia and Micronutrient Supplementation in the AppNL–G–F Model of Alzheimer’s Disease
Source: Front Aging Neurosci. 2022 Apr 26;14:876826. doi: 10.3389/fnagi.2022.876826 (PMC9094364; doi:10.3389/fnagi.2022.876826)
Supplement: Supplementary file 1 [file Data_Sheet_1.docx]

Supplementary Results

## Barnes Maze

In the first part of the Barnes maze test (acquisition phase), spatial learning and memory, expressed as the latency the mice needed to locate the target hole, was tested twice a day, with a short interim period of 15 minutes. Mean values are illustrated for each of the four training days in Figure S1. Testing for statistically significant effects, which was performed on day 4, did not reveal differences between experimental groups. After applying reversal settings in time block 2 and 3, we additionally evaluated latencies to the previous target position on day 1 each time in order to examine potential differences in long-term memory of the mice to their respective target at former time points. However, no effects were detected for any group or time block (data not shown).


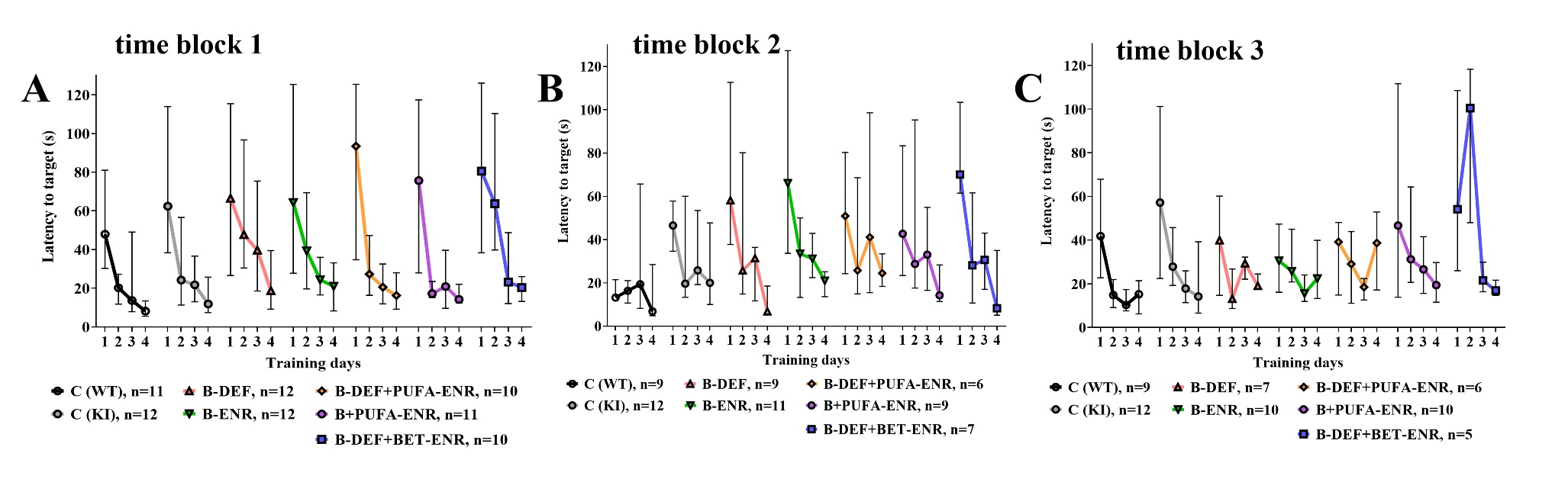


**Supplementary Figure 1 (S1).** Barnes maze acquisition phase: (A-C) latency to target hole in time block 1-3 (4 subsequent days of training each time: average of 2 sessions per day); statistical testing for differences on day 4; median ± IQR; outliers beyond threefold IQR removed; p < 0.05 (Mann-Whitney-U-test) considered statistically significant (*).

In the second part of the test (probe trial, day 5), memory performance was tested by measuring the latency to the target hole in all three time blocks (Figure S2 A-C). Additionally, the time the mice spent in the target quadrant of the Barnes maze arena was determined (Figure S2 D-F). In time block 2, hyperhomocysteinemic mice supplemented with PUFA needed significantly longer to reach the target position that had previously been learned in the acquisition phase (p=0.013). However, this finding might be biased by an unknown factor, since the effect was not consistently detected in the other time blocks and therefore, appears to be biologically irrelevant. An impact of the AD-like pathology or other dietary interventions on spatial memory was not observed in this test. Also, the cumulative time at target, meaning the persistence of a mouse at the position where the escape box had been located during the acquisition phase, did not significantly differ between the groups.

**
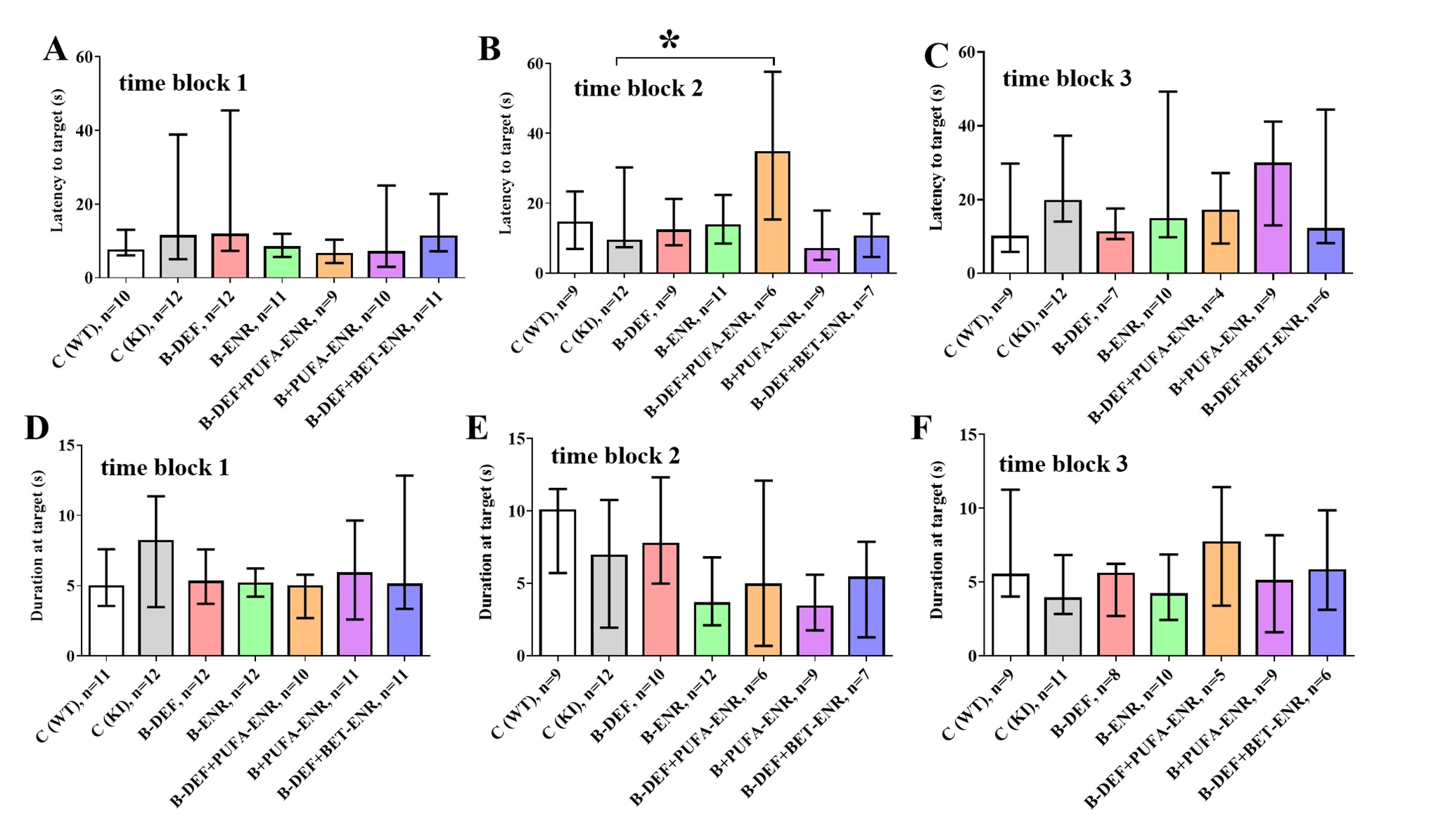
**

**Supplementary Figure 2 (S2).** Barnes maze probe trial in time block 1-3: (A-C) latency to target hole and (D-F) cumulative duration at target hole; median ± IQR; outliers beyond threefold IQR removed; p < 0.05 (Mann-Whitney-U-test) considered statistically significant (*).

## Y-Maze

Working memory was assessed in the Y-maze (Figure S3) by measuring the spontaneous alternation of the mice, a natural behavior that is hypothesized to be disrupted as a consequence of cognitive deterioration (Equation 2). In time block 2, for unknown reasons, the KI control mice performed poorly, with the result that comparisons with some of the dietary treatment groups became significant (B-DEF p=0.040; B-ENR p=0.028; B-DEF+BET-ENR p=0.026). It has to be emphasized that these effects were not sustained and should therefore not be overestimated. Overall, no consistent effects were related to either the AD-like genotype or treatment with different experimental diets.

**
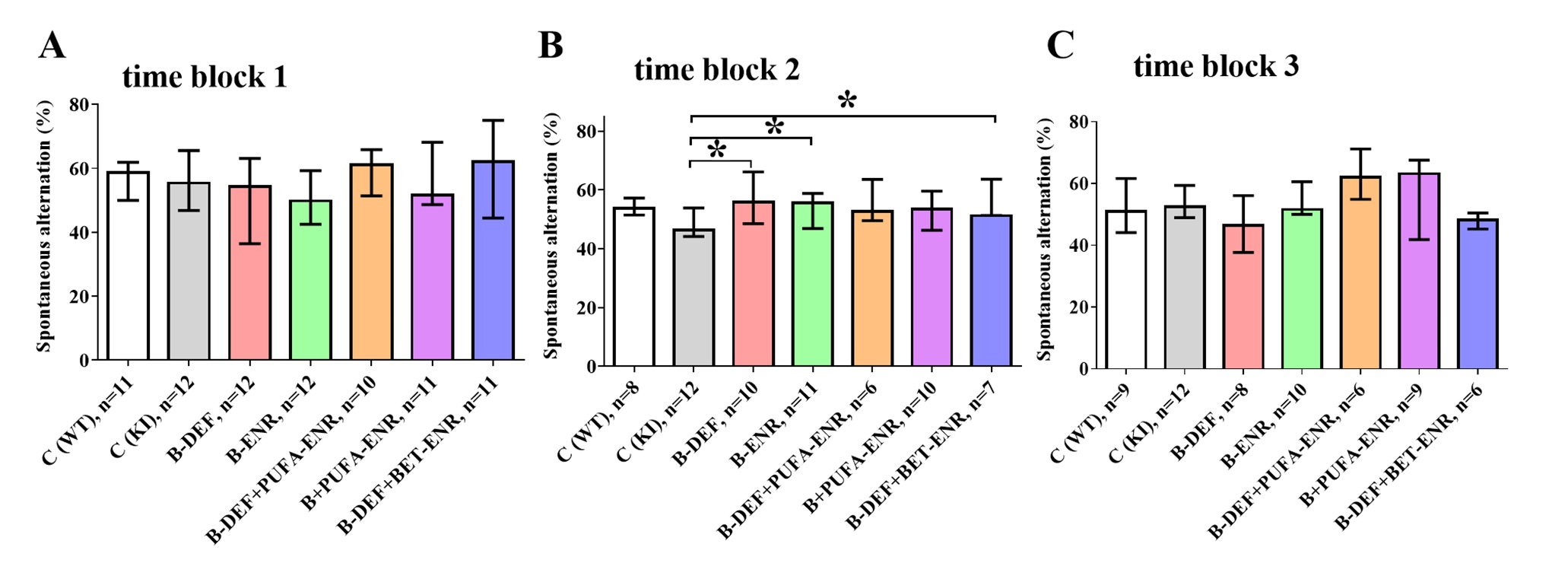
**

**Supplementary Figure 3 (S3).** Y-maze, spontaneous alternation rate in time block 1-3 (A-C); median ± IQR; outliers beyond threefold IQR removed; p < 0.05 (Mann-Whitney-U-test) considered statistically significant (*).

**
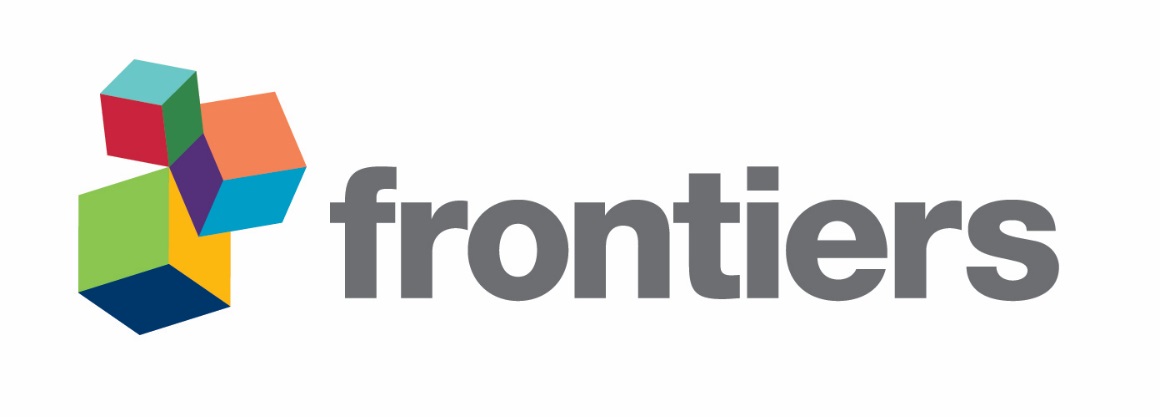
**
