# Supplementary material for: The Roles of Long-Term Hyperhomocysteinemia and Micronutrient Supplementation in the AppNL–G–F Model of Alzheimer’s Disease
Source: Front Aging Neurosci. 2022 Apr 26;14:876826. doi: 10.3389/fnagi.2022.876826 (PMC9094364; doi:10.3389/fnagi.2022.876826)
Supplement: Supplementary file 2 [file Table_1.docx]

Supplementary Table 1

**Supplementary Table 1.** Experimental diets

|  | **Control** | **B-DEF** | **B-ENR** | **B-DEF +PUFA-ENR** | **B**  **+PUFA-ENR** | **B-DEF +BET-ENR** |
| --- | --- | --- | --- | --- | --- | --- |
| Casein | 140.0 | 140.0 | 140.0 | 140.0 | 140.0 | 140.0 |
| Corn starch | 355.66 | 345.69 | 355.48 | 345.69 | 355.48 | 335.69 |
| Maltodextrin | 155.0 | 155.0 | 155.0 | 155.0 | 155.0 | 155.0 |
| Sucrose | 100.0 | 100.0 | 100.0 | 100.0 | 100.0 | 100.0 |
| Dextrose | 100.0 | 100.0 | 100.0 | 100.0 | 100.0 | 100.0 |
| Cellulose | 50.0 | 50.0 | 50.0 | 50.0 | 50.0 | 50.0 |
| Mineral premix | 35.0 | 35.0 | 35.0 | 35.0 | 35.0 | 35.0 |
| Vitamin pre-mix  (w/o B-vitamins) | 10.0 | 10.0 | 10.0 | 10.0 | 10.0 | 10.0 |
| Soybean oil | 19.0 | 19.0 | 19.0 | —— | —— | 19.0 |
| Coconut oil | 9.0 | 9.0 | 9.0 | 11.3 | 11.3 | 9.0 |
| Corn oil | 22.0 | 22.0 | 22.0 | 18.7 | 18.7 | 22.0 |
| Fish oil (eicosapentaenoic acid/docosahexaenoic acid = 1:4) | —— | —— | —— | 20.0 | 20.0 | —— |
| L-Cystine | 1.8 | 1.8 | 1.8 | 1.8 | 1.8 | 1.8 |
| Tert-butylhydroquinone | 0.008 | 0.008 | 0.008 | 0.008 | 0.008 | 0.008 |
| Choline  bitartrate, 41% | 2.50 | 2.50 | 2.50 | 2.50 | 2.50 | 2.50 |
| Pyridoxine-HCl  (Vit. B6) | 0.007 | —— | 0.100 | —— | 0.100 | —— |
| Cyanocobalamin, 0.1% (Vit. B12) | 0.025 | —— | 0.100 | —— | 0.100 | —— |
| Folic acid, 80% | 0.0025 | —— | 0.0125 | —— | 0.0125 | —— |
| Sulfathiazole sodium | —— | 10.0 | —— | 10.0 | —— | 10.0 |
| Betaine | —— | —— | —— | —— | —— | 10.0 |
| Sum | 1000 | 1000 | 1000 | 1000 | 1000 | 1000 |
