# Supplementary material for: The Roles of Long-Term Hyperhomocysteinemia and Micronutrient Supplementation in the AppNL–G–F Model of Alzheimer’s Disease
Source: Front Aging Neurosci. 2022 Apr 26;14:876826. doi: 10.3389/fnagi.2022.876826 (PMC9094364; doi:10.3389/fnagi.2022.876826)
Supplement: Supplementary file 3 [file Table_2.docx]

Supplementary Table 2

**Supplementary Table 2.** Further information on reduced numbers of observations (n) in the experiments; (a) no value obtained by LC-MS/MS analysis; (b) sampling of appropriate volumes of uncoagulated whole blood failed; (c) total "n" was different in the proteome analysis.

| **Figure** | **Section** | **Group** | **N** | **Animal Loss** | **Outliers** | **Others (a,b,c)** |
| --- | --- | --- | --- | --- | --- | --- |
| 3 | A | 1 | 12 | 0 | 0 |  |
|  |  | 2 | 10 | 0 | 1 | 1 (a) |
|  |  | 3 | 12 | 0 | 0 |  |
|  |  | 4 | 12 | 0 | 0 |  |
|  |  | 5 | 11 | 1 | 0 |  |
|  |  | 6 | 12 | 0 | 0 |  |
|  |  | 7 | 12 | 0 | 0 |  |
|  | B | 1 | 9 | 3 | 0 |  |
|  |  | 2 | 12 | 0 | 0 |  |
|  |  | 3 | 10 | 2 | 0 |  |
|  |  | 4 | 12 | 0 | 0 |  |
|  |  | 5 | 8 | 4 | 0 |  |
|  |  | 6 | 9 | 1 | 2 |  |
|  |  | 7 | 7 | 4 | 0 | 1 (a) |
|  | C | 1 | 9 | 3 | 0 |  |
|  |  | 2 | 11 | 0 | 0 | 1 (a) |
|  |  | 3 | 8 | 4 | 0 |  |
|  |  | 4 | 11 | 1 | 0 |  |
|  |  | 5 | 6 | 6 | 0 |  |
|  |  | 6 | 10 | 2 | 0 |  |
|  |  | 7 | 6 | 6 | 0 |  |
|  | D | 1 | 7 | 3 | 2 |  |
|  |  | 2 | 12 | 0 | 0 |  |
|  |  | 3 | 8 | 4 | 0 | 1 (a) |
|  |  | 4 | 10 | 2 | 0 |  |
|  |  | 5 | 6 | 6 | 0 |  |
|  |  | 6 | 8 | 3 | 1 |  |
|  |  | 7 | 6 | 6 | 0 |  |
|  | E | 1 | 12 | 0 | 0 |  |
|  |  | 2 | 12 | 0 | 0 |  |
|  |  | 3 | 12 | 0 | 0 |  |
|  |  | 4 | 12 | 0 | 0 |  |
|  |  | 5 | 11 | 1 | 0 |  |
|  |  | 6 | 11 | 0 | 0 | 1 (a) |
|  |  | 7 | 12 | 0 | 0 |  |
|  | F | 1 | 9 | 3 | 0 |  |
|  |  | 2 | 12 | 0 | 0 |  |
|  |  | 3 | 10 | 2 | 0 |  |
|  |  | 4 | 11 | 0 | 0 | 1 (a) |
|  |  | 5 | 8 | 4 | 0 |  |
|  |  | 6 | 11 | 1 | 0 |  |
|  |  | 7 | 8 | 4 | 0 |  |
|  | G | 1 | 8 | 3 | 0 | 1 (a) |
|  |  | 2 | 11 | 0 | 0 | 1 (a) |
|  |  | 3 | 8 | 4 | 0 |  |
|  |  | 4 | 7 | 2 | 0 | 3 (a) |
|  |  | 5 | 6 | 6 | 0 |  |
|  |  | 6 | 8 | 3 | 0 | 1 (a) |
|  |  | 7 | 6 | 6 | 0 |  |
| 4 | A | 1 | 11 | 1 | 0 |  |
|  |  | 2 | 12 | 0 | 0 |  |
|  |  | 3 | 12 | 0 | 0 |  |
|  |  | 4 | 12 | 0 | 0 |  |
|  |  | 5 | 11 | 1 | 0 |  |
|  |  | 6 | 12 | 0 | 0 |  |
|  |  | 7 | 12 | 0 | 0 |  |
|  | B | 1 | 11 | 1 | 0 |  |
|  |  | 2 | 12 | 0 | 0 |  |
|  |  | 3 | 12 | 0 | 0 |  |
|  |  | 4 | 12 | 0 | 0 |  |
|  |  | 5 | 11 | 1 | 0 |  |
|  |  | 6 | 12 | 0 | 0 |  |
|  |  | 7 | 12 | 0 | 0 |  |
|  | C | 1 | 8 | 3 | 1 |  |
|  |  | 2 | 12 | 0 | 0 |  |
|  |  | 3 | 10 | 2 | 0 |  |
|  |  | 4 | 12 | 0 | 0 |  |
|  |  | 5 | 7 | 5 | 0 |  |
|  |  | 6 | 11 | 1 | 0 |  |
|  |  | 7 | 7 | 5 | 0 |  |
|  | D | 1 | 9 | 3 | 0 |  |
|  |  | 2 | 12 | 0 | 0 |  |
|  |  | 3 | 10 | 2 | 0 |  |
|  |  | 4 | 12 | 0 | 0 |  |
|  |  | 5 | 6 | 5 | 1 |  |
|  |  | 6 | 11 | 1 | 0 |  |
|  |  | 7 | 7 | 5 | 0 |  |
|  | E | 1 | 9 | 3 | 0 |  |
|  |  | 2 | 12 | 0 | 0 |  |
|  |  | 3 | 8 | 4 | 0 |  |
|  |  | 4 | 11 | 1 | 0 |  |
|  |  | 5 | 6 | 6 | 0 |  |
|  |  | 6 | 10 | 2 | 0 |  |
|  |  | 7 | 6 | 6 | 0 |  |
|  | F | 1 | 9 | 3 | 0 |  |
|  |  | 2 | 12 | 0 | 0 |  |
|  |  | 3 | 8 | 4 | 0 |  |
|  |  | 4 | 11 | 1 | 0 |  |
|  |  | 5 | 6 | 6 | 0 |  |
|  |  | 6 | 9 | 2 | 1 |  |
|  |  | 7 | 6 | 6 | 0 |  |
| 5 | A | 1 | 9 | 3 | 0 |  |
|  |  | 2 | 12 | 0 | 0 |  |
|  |  | 3 | 6 | 4 | 0 | 2 (b) |
|  |  | 4 | 8 | 2 | 1 | 1 (b) |
|  |  | 5 | 5 | 6 | 0 | 1 (b) |
|  |  | 6 | 9 | 3 | 0 |  |
|  |  | 7 | 4 | 6 | 1 | 1 (b) |
|  | B | 1 | 8 | 3 | 1 |  |
|  |  | 2 | 12 | 0 | 0 |  |
|  |  | 3 | 6 | 4 | 0 | 2 (b) |
|  |  | 4 | 8 | 2 | 1 | 1 (b) |
|  |  | 5 | 5 | 6 | 0 | 1 (b) |
|  |  | 6 | 9 | 3 | 0 |  |
|  |  | 7 | 5 | 6 | 0 | 1 (b) |
|  | C | 1 | 8 | 3 | 1 |  |
|  |  | 2 | 11 | 0 | 1 |  |
|  |  | 3 | 6 | 4 | 0 | 2 (b) |
|  |  | 4 | 8 | 2 | 1 | 1 (b) |
|  |  | 5 | 5 | 6 | 0 | 1 (b) |
|  |  | 6 | 9 | 3 | 0 |  |
|  |  | 7 | 5 | 6 | 0 | 1 (b) |
| 7 | A_1 | 1 | 8 | 3 | 0 | c |
|  |  | 2 | 11 | 0 | 0 | c |
|  | A_2 | 1 | 8 | 3 | 0 | c |
|  |  | 2 | 11 | 0 | 0 | c |
|  | A_3 | 1 | 8 | 3 | 0 | c |
|  |  | 2 | 11 | 0 | 0 | c |
|  | A_4 | 1 | 8 | 3 | 0 | c |
|  |  | 2 | 10 | 0 | 1 | c |
|  | A_5 | 1 | 7 | 3 | 1 | c |
|  |  | 2 | 10 | 0 | 1 | c |
|  | A_6 | 1 | 7 | 3 | 1 | c |
|  |  | 2 | 11 | 0 | 0 | c |
|  | B_1 | 2 | 11 | 0 | 0 | c |
|  |  | 3 | 8 | 4 | 0 | c |
|  | B_2 | 2 | 11 | 0 | 0 | c |
|  |  | 3 | 8 | 4 | 0 | c |
|  | B_3 | 2 | 11 | 0 | 0 | c |
|  |  | 3 | 8 | 4 | 0 | c |
|  | B_4 | 2 | 11 | 0 | 0 | c |
|  |  | 3 | 8 | 4 | 0 | c |
|  | B_5 | 2 | 11 | 0 | 0 | c |
|  |  | 3 | 8 | 4 | 0 | c |
|  | B_6 | 2 | 11 | 0 | 0 | c |
|  |  | 3 | 8 | 4 | 0 | c |
| 8 | - | 1 | 8 | 3 | 1 |  |
|  |  | 2 | 12 | 0 | 0 |  |
|  |  | 3 | 8 | 4 | 0 |  |
|  |  | 4 | 10 | 2 | 0 |  |
|  |  | 5 | 6 | 6 | 0 |  |
|  |  | 6 | 8 | 3 | 1 |  |
|  |  | 7 | 5 | 6 | 1 |  |
| A 1 | A | 1 | 11 | 1 | 0 |  |
|  |  | 2 | 12 | 0 | 0 |  |
|  |  | 3 | 12 | 0 | 0 |  |
|  |  | 4 | 12 | 0 | 0 |  |
|  |  | 5 | 10 | 1 | 1 |  |
|  |  | 6 | 11 | 1 | 0 |  |
|  |  | 7 | 10 | 0 | 2 |  |
|  | B | 1 | 9 | 3 | 0 |  |
|  |  | 2 | 12 | 0 | 0 |  |
|  |  | 3 | 9 | 2 | 1 |  |
|  |  | 4 | 11 | 0 | 1 |  |
|  |  | 5 | 6 | 6 | 0 |  |
|  |  | 6 | 9 | 2 | 1 |  |
|  |  | 7 | 7 | 5 | 0 |  |
|  | C | 1 | 9 | 3 | 0 |  |
|  |  | 2 | 12 | 0 | 0 |  |
|  |  | 3 | 7 | 4 | 1 |  |
|  |  | 4 | 10 | 2 | 0 |  |
|  |  | 5 | 6 | 6 | 0 |  |
|  |  | 6 | 10 | 2 | 0 |  |
|  |  | 7 | 5 | 6 | 1 |  |
| A 2 | A | 1 | 10 | 1 | 1 |  |
|  |  | 2 | 12 | 0 | 0 |  |
|  |  | 3 | 12 | 0 | 0 |  |
|  |  | 4 | 11 | 0 | 1 |  |
|  |  | 5 | 9 | 2 | 1 |  |
|  |  | 6 | 10 | 1 | 1 |  |
|  |  | 7 | 11 | 1 | 0 |  |
|  | B | 1 | 11 | 1 | 0 |  |
|  |  | 2 | 12 | 0 | 0 |  |
|  |  | 3 | 12 | 0 | 0 |  |
|  |  | 4 | 12 | 0 | 0 |  |
|  |  | 5 | 10 | 2 | 0 |  |
|  |  | 6 | 11 | 1 | 0 |  |
|  |  | 7 | 11 | 1 | 0 |  |
|  | C | 1 | 9 | 3 | 0 |  |
|  |  | 2 | 12 | 0 | 0 |  |
|  |  | 3 | 9 | 2 | 1 |  |
|  |  | 4 | 11 | 0 | 1 |  |
|  |  | 5 | 6 | 6 | 0 |  |
|  |  | 6 | 9 | 2 | 1 |  |
|  |  | 7 | 7 | 5 | 0 |  |
|  | D | 1 | 9 | 3 | 0 |  |
|  |  | 2 | 12 | 0 | 0 |  |
|  |  | 3 | 10 | 2 | 0 |  |
|  |  | 4 | 12 | 0 | 0 |  |
|  |  | 5 | 6 | 6 | 0 |  |
|  |  | 6 | 9 | 2 | 1 |  |
|  |  | 7 | 7 | 5 | 0 |  |
|  | E | 1 | 9 | 3 | 0 |  |
|  |  | 2 | 12 | 0 | 0 |  |
|  |  | 3 | 7 | 4 | 1 |  |
|  |  | 4 | 10 | 2 | 0 |  |
|  |  | 5 | 4 | 6 | 2 |  |
|  |  | 6 | 9 | 3 | 0 |  |
|  |  | 7 | 6 | 6 | 0 |  |
|  | F | 1 | 9 | 3 | 0 |  |
|  |  | 2 | 11 | 0 | 1 |  |
|  |  | 3 | 8 | 4 | 0 |  |
|  |  | 4 | 10 | 2 | 0 |  |
|  |  | 5 | 5 | 6 | 1 |  |
|  |  | 6 | 9 | 3 | 0 |  |
|  |  | 7 | 6 | 6 | 0 |  |
| A 3 | A | 1 | 11 | 1 | 0 |  |
|  |  | 2 | 12 | 0 | 0 |  |
|  |  | 3 | 12 | 0 | 0 |  |
|  |  | 4 | 12 | 0 | 0 |  |
|  |  | 5 | 10 | 2 | 0 |  |
|  |  | 6 | 11 | 1 | 0 |  |
|  |  | 7 | 11 | 1 | 0 |  |
|  | B | 1 | 8 | 3 | 1 |  |
|  |  | 2 | 12 | 0 | 0 |  |
|  |  | 3 | 10 | 2 | 0 |  |
|  |  | 4 | 11 | 1 | 0 |  |
|  |  | 5 | 6 | 6 | 0 |  |
|  |  | 6 | 10 | 2 | 0 |  |
|  |  | 7 | 7 | 5 | 0 |  |
|  | C | 1 | 9 | 3 | 0 |  |
|  |  | 2 | 12 | 0 | 0 |  |
|  |  | 3 | 8 | 4 | 0 |  |
|  |  | 4 | 10 | 2 | 0 |  |
|  |  | 5 | 6 | 6 | 0 |  |
|  |  | 6 | 9 | 3 | 0 |  |
|  |  | 7 | 6 | 6 | 0 |  |

**
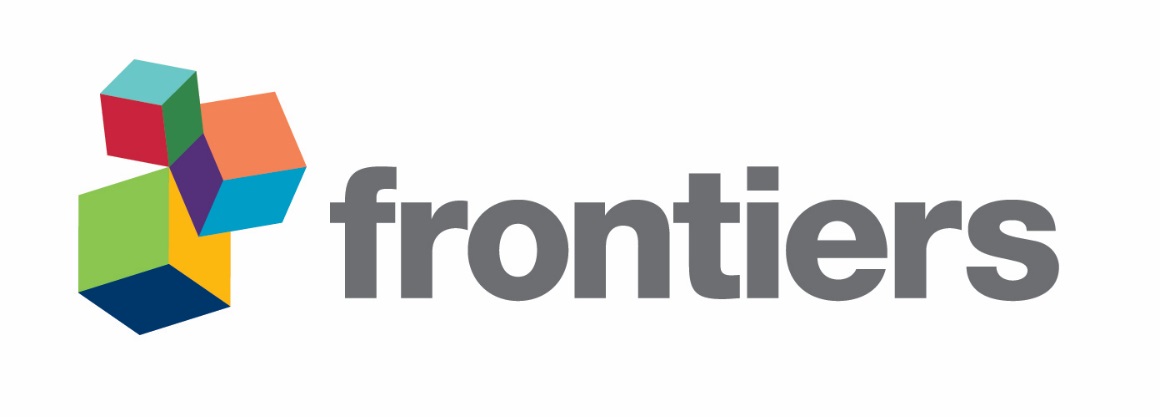
**
